# Analysis of Porcine RIG-I Like Receptors Revealed the Positive Regulation of RIG-I and MDA5 by LGP2

**DOI:** 10.3389/fimmu.2021.609543

**Published:** 2021-05-18

**Authors:** Shuangjie Li, Jie Yang, Yuanyuan Zhu, Hui Wang, Xingyu Ji, Jia Luo, Qi Shao, Yulin Xu, Xueliang Liu, Wanglong Zheng, François Meurens, Nanhua Chen, Jianzhong Zhu

**Affiliations:** ^1^ Comparative Medicine Research Institute, Yangzhou University, Yangzhou, China; ^2^ College Veterinary Medicine, Yangzhou University, Yangzhou, China; ^3^ Joint International Research Laboratory of Agriculture and Agri-Product Safety, Yangzhou, China; ^4^ Jiangsu Co-innovation Center for Prevention and Control of Important Animal Infectious Diseases and Zoonoses, Yangzhou, China; ^5^ INRAE, Oniris, BIOEPAR, Nantes, France; ^6^ Department of Veterinary Microbiology and Immunology, Western College of Veterinary Medicine, University of Saskatchewan, Saskatoon, SK, Canada

**Keywords:** RIG-like receptors (RLRs), porcine, LGP2, positive regulation, species specificity

## Abstract

The RLRs play critical roles in sensing and fighting viral infections especially RNA virus infections. Despite the extensive studies on RLRs in humans and mice, there is a lack of systemic investigation of livestock animal RLRs. In this study, we characterized the porcine RLR members RIG-I, MDA5 and LGP2. Compared with their human counterparts, porcine RIG-I and MDA5 exhibited similar signaling activity to distinct dsRNA and viruses, *via* similar and cooperative recognitions. Porcine LGP2, without signaling activity, was found to positively regulate porcine RIG-I and MDA5 in transfected porcine alveolar macrophages (PAMs), gene knockout PAMs and PK-15 cells. Mechanistically, LGP2 interacts with RIG-I and MDA5 upon cell activation, and promotes the binding of dsRNA ligand by MDA5 as well as RIG-I. Accordingly, porcine LGP2 exerted broad antiviral functions. Intriguingly, we found that porcine LGP2 mutants with defects in ATPase and/or dsRNA binding present constitutive activity which are likely through RIG-I and MDA5. Our work provided significant insights into porcine innate immunity, species specificity and immune biology.

## Introduction

The innate immune system, as the first line of host defense, relies on the pattern recognition receptors (PRRs) to sense various danger signals from pathogens *via* detecting the pathogen associated molecular patterns (PAMPs) (1). Additionally, the host damage associated molecular patterns (DAMPs) are also recognized by PRRs to maintain homeostasis (1, 2). PRRs encompass Toll-like receptors (TLRs), RIG-I-like receptors (RLRs), NOD-like receptors (NLRs), C-type lectin like receptors (CLRs) and cytosolic DNA receptors (CDRs). Upon activation by PAMPs and DAMPs, the PRRs trigger intracellular signaling, which induces either downstream gene transcription or protease-dependent cytokine secretion. Thus, the produced anti-viral interferons (IFNs), proinflammatory cytokines and chemokines will directly combat pathogens and shape subsequent adaptive immunity.

RLRs are the family of DExD/H-box helicases and the main cytosolic viral RNA sensors including homologous RIG-I, MDA5 and LGP2 (3). The prototypic RIG-I as well as MDA5 were discovered more than 15 years ago in overexpression studies to induce type I interferons (IFNs) (4, 5). Both RIG-I and MDA5 have similar domain architecture: N-terminal signal active two tandem caspase recruitment domain (CARDs), middle DExD/H-box helicase domain and C-terminal repressor domain (CTD) or regulatory domain (RD) (6, 7). The effective RNA recognized by RIG-I is the blunt ended 5’-ppp dsRNA (double stranded RNA) with the 5′-terminal nucleotide 2’-O-unmethylated, whereas MDA5 prefers internal binding to the long dsRNA with no end specificity (8, 9). Upon recognition of dsRNA, RIG-I and MDA5 activate downstream common adaptor MAVS, which recruit TRAF3/TBK1/IKKε and TRAF6/IKKs to activate transcription factors IRF3 and NF-κB, driving IFN and proinflammatory cytokine expression, respectively (10).

The third member of RLRs is LGP2, with similar helicase and CTD domains as RIG-I and MDA5, but due to lack of N terminal CARDs, has no signaling activity. Instead, it is able to regulate RIG-I and MDA5 signaling likely *via* its strong capability of binding dsRNA (11). It was initially shown to inhibit RIG-I and MDA5 mediated signaling through multiple possible mechanisms (12–14). Later on, using the LGP2 gene KO mice, the LGP2 was suggested to positively regulate RIG-I and MDA5 signaling (15, 16). Further studies tried to reconcile the controversial regulatory roles of LGP2 by proposing that LGP2 is a concentration dependent biphasic switch: at low concentration when cells are in steady state, LGP2 enhances MDA5 activation to help fight infection, whereas at high concentration in late infection stages, LGP2 exerts a general inhibition to RIG-I and MDA5 signaling to resolve the inflammation (17, 18). Nevertheless, the exact role of LGP2 in the regulation of RIG-I/MDA5 and in antiviral signaling still remains an open question (8, 19).

RLRs are expressed almost in all mammalian cell types, and play key roles in virus recognition and immune responses (6, 20). RIG-I and MDA5 play independent and synergistic roles in sensing different RNA viruses (7, 21). Consistent with the short RNA agonists, RIG-I mainly senses most negative-sense single-stranded RNA viruses which generate short 5’-ppp dsRNA during replication. These viruses include *Filoviridae* (Ebola virus), *Paramyxoviridae* (Measle virus, Sendai virus and Newcastle disease virus), *Pneumoviridae* (Respiratory syncytial virus), *Orthomyxoviridae* (Influenza virus), *Bunyaviridae* (Hantavirus), *Rhabdoviridae* (Vesicular stomatitis virus and Rabies virus). RIG-I also senses positive-sense single-stranded RNA viruses such as *Flaviviridae* (Hepatitis C virus and Japanese encephalitis virus). In contrast, MDA5 recognizes long dsRNA and thus senses the positive-sense single-stranded RNA viruses such as the *Picornaviridae* (Encephalomyocarditis virus, Poliovirus, Coxasackie virus, and Arteriviruses as well as Hepatitis D virus). On the other hand, both RIG-I and MDA5 cross-detect the same viruses including double-stranded RNA *Reoviridae* (Rotavirus), positive-sense single-stranded *Flaviviridae* (Dengue virus and West Nile virus), *Coronaviridae* (Murine hepatitis virus).

In addition to RNA viruses mentioned above, RLRs are also implicated in other pathogen recognitions. For example, RIG-I is able to sense some DNA viruses such as Adenovirus, Poxvirus and Herpesvirus because these viruses produce short dsRNA through the type III RNA polymerase during replication. Other reports also showed that RIG-I can detect the RNA from bacteria such as *Listeria monocytogenes*, *Helicobacter pylori* and *Shigella flexneri* (22). However, till now, there is a lacking of systemic investigation on RLRs of animal source including *Sus scrofa* (pig). Porcine (pig) is an important livestock species but also a promising animal model to study several human diseases including infectious diseases (23, 24). Accumulating evidences showed the essential roles of RLRs in the antiviral function against a number of porcine viruses such as porcine reproductive and respiratory syndrome virus (PRRSV) (25), porcine epidemic diarrhea virus (PEDV) (26), classical swine fever virus (CSFV) (27), porcine circovirus type 2 (PCV2) (28), porcine deltacoronavirus (PDCoV) (29), seneca valley virus (SVV) (30) *etc.* Further, the interrelationship between RLR members is still not completely resolved. In this study, we characterized the porcine RIG-I, MDA5 and LGP2, and analyzed their mutual relationship. We found that porcine RIG-I and MDA5 exhibit similar and cooperative functions in recognition of RNA and sensing viruses; porcine LGP2 positively regulates RIG-I and MDA5 by promoting the RNA recognition; and interestingly, the ATPase sites in porcine LGP2 play unique roles in RIG-I and MDA5 signaling.

## Materials and Methods

### Cells and Reagents

HEK293T, Vero cells, Meat animal research center-145 (Marc-145), Intestinal porcine enterocyte cell line (IPEC-J2) and porcine kidney-15 (PK-15, ATCC CCL-33™) cells were cultured in DMEM (Hyclone Laboratories, USA) containing 10% fetal bovine serum (FBS) and 100 IU/ml of penicillin plus 100 μg/ml streptomycin. Porcine alveolar macrophages (PAMs, 3D4/21, ATCC CRL-2843™) were cultured in RPMI (Hyclone Laboratories) containing 10% FBS with penicillin/streptomycin. All cells were maintained at 37°C with 5% CO_2_ in a humidified incubator. TRIpure Reagent for RNA extraction was from Aidlab (Beijing, China). PfuUltra II Fusion HS DNA polymerase was from Agilent Technologies (Beijing, China). Restriction endonucleases and T4 DNA ligase (M0203S) were purchased from New England Biolabs (Beijing, China). HiScript^®^ 1st Strand cDNA Synthesis Kit and ChamQ Universal SYBR qPCR Master Mix, 2×Taq Master Mix (Dye plus), PCR Purification Kit, Gel Extraction Kit, Plasmid Mini-prep Kit were from Vazyme Biotech Co.,Ltd (Nanjing, China). Gateway^®^ LR Clonase™ II Enzyme mix, Goat Anti-Rabbit IgG DyLight 488 (SA244803), Goat Anti-Mouse IgG DyLight 594 (SD251085), Lipofectamine™ 2000, Lipofectamine™ 3000 and streptavidin agarose were from ThermoFisher Scientific (Shanghai, China). Donkey Anti-Rabbit IgG Alexa Fluor 647 (ab150075) and Goat Anti-Mouse IgG H&L Alexa Fluor^®^ 594 (ab150120) were from Abcam (Shanghai, China). TransIT-LT1 Transfection Reagent was purchased from Mirus Bio (Madison, USA). BluePlus Protein Marker, anti-HA mouse mAb (HT301), anti-FLAG mouse mAb (HT201), anti-GFP mouse mAb (HT801), anti-Actin mouse mAb (HC201), HRP anti-mouse IgG (HS201), HRP anti-rabbit IgG (HS101), Fast Mutagenesis System Kit and TransDetect Double-Luciferase Reporter Assay Kit were bought from Transgen Biotech (Beijing, China). The anti-HA (C29F4) rabbit pAb (3724S), anti-p-TBK1 (D52C2) (5483S), anti-p-IRF3 (4D4G) (29047S) and anti-IRF3 (D614C) (11904T) were from Cell Signaling Technology (Danvers, MA, US). Poly (I:C)-LMW, poly (I:C)-HMW, and poly (I:C) HMW Biotin were purchased from InvivoGen (San Diego, California, USA). Protein A/G PLUS-Agarose and the anti- Influenza A NS1 mouse mAb (sc-130568) was purchased from Santa Cruz Biotechnology (Dallas, Texas, USA). Protino^®^ Ni-TED 2000 Packed Columns was from Macherey-Nagel (Düren, Germany). Micro Protein PAGE Recovery Kit was from Sangon Biotech (Shanghai, China). HSV1-GFP, VSV-GFP, SeV-GFP, EMCV were used as we previously reported (31). Influenza type A virus (IAV) PR8 H1N1 and Swine IAV (SwIAV) H9N2 were provided by Dr. Hongjun Chen at Shanghai Veterinary Research Institute China. Porcine epidemic diarrhea virus (PEDV) strain YC2014 was from Dr. Hongjie Fan in Nanjing Agricultural University China. Porcine reproductive and respiratory syndrome virus (PRRSV) strain JXA1-R and anti-N rabbit antibody were preserved in our laboratory (32).

### Molecular Cloning and Gene Mutations

Total RNA was extracted from PAMs or PK15 using TRIpure Reagent. From the total RNA, the porcine open reading frames (ORFs) of RIG-I, MDA5 and LGP2 were amplified by RT-PCR using the designed primers shown in **Supplementary Table 1**. The PCR products were digested with *Sal*I/*Sma*I, *Sal*I/*Sma*I and *Sal*I/*EcoR*V, respectively, and cloned into the *Sal*I/*EcoR*V sites of the Gateway entry vector pENTR4-2HA or pENTR4-3FLAG which carries 2HA/3FLAG tag to express C-terminal HA or FLAG tagged proteins. The sequence confirmed HA-tagged and FLAG-tagged pRIG-I, pMDA5 and pLGP2 were all transferred from pENTR4 vectors to Destination vectors pDEST47 (Addgene) by LR recombination to obtain the final pcDNA recombinant expression vectors. Expression plasmids pcDNA human RIG-I-FLAG, pcDNA human MDA5-FLAG and pEGFP-C1-pMAVS were previously cloned and stored in our laboratory. If not specifically mentioned, the LGP2, RIG-I and MDA5 refer to those of porcine origin.

For LGP2 subcloning and mutations, the helicase ATP binding domain (L1), Δhelicase ATP binding domain (ΔL1), helicase CTER domain (L2), CTR domain (L3) and ΔCTR domain (ΔL3) fragments were amplified by PCR using PfuUltra II Fusion HS DNA Polymerase from pcDNA pLGP2-HA plasmid template using the designed primer pairs shown in **Supplementary Table 1**. For LGP2 deletion mutant Δhelicase CTER (ΔL2), both fragments flanking the deletion site were amplified by PCR from LGP2 plasmid respectively; next, the two flanking fragments together with the Bridge fragment (Fusion primer) as the templates were joined together by fusion PCR using the designed primer pair L1F/L3R shown in **Supplementary Table 1**. The LGP2 L1, ΔL1, L2, ΔL2, L3 and ΔL3 PCR products digested with *Nhe*I and *EcoR*V were cloned into the same sites of pcDNA vector expressing C-terminal 2HA as we described previously (33). The mutants of LGP2 M1 K30A, MIIa K138E/Y142F, MIII T167A/S169A, and K654E were produced by mutation PCR using pcDNA pLGP2-HA as a template and the Fast Mutagenesis System Kit. The mutation PCR primer pairs were shown in **Supplementary Table 1**.

### CRISPR gRNA Mediated Stable KO Cells and Homozygous KO PAM Cell Clones

The CRISPR gRNAs targeting porcine RIG-I, MDA5 and LGP2 were designed using the web tool from Benchling (www.benchling.com). For porcine RIG-I, MDA5 and LGP2, four, three and three gRNAs were chosen, respectively, based on the predicted high scores. The encoding DNA sequences are shown in **Supplementary Table 2**. The annealed gRNA encoding DNA pairs were ligated with *Bsm*BI digested lentiCRISPRv2 vector (Addgene), and the efficiency and specificity of these gRNA expressing lentiviral vectors targeting porcine RIG-I, MDA5 and LGP2 were demonstrated as shown in **Supplementary Figure 2**. The effective gRNAs are gRNAs 2 and 4 for pRIG-I, the gRNAs 1, 2 and 3 for pMDA5, and gRNAs 1, 2 and 3 for pLGP2. The gRNA expressing lentiviruses were generated by co-transfecting each effective lentiCRISPRv2-gRNAs with package plasmids psPAX2 and pMD2.G into 293T cells using Lipofectamine 2000. For RIG-I, MDA5 or LGP2, the supernatants containing different gRNA expressing lentiviruses were mixed equally and used to infect the PAMs or PK-15 cells, then the infected cells were selected with 1 μg/ml puromycin. After puromycin selection, the CRISPR vector control, RIG-I KO, MDA5 KO and pLGP2 KO stable PAMs and PK-15 cells were obtained and ready for subsequent experiments.

The RIG-I KO, MDA5 KO and LGP2 KO stable PAMs were subcloned by limited dilution and monoclonal cells were chosen. The individual grown cell clones were screened by RT-PCR using the designed primers shown in **Supplementary Table 2**: First, the PCR products were run by non-denatured polyacrylamide gel electrophoresis and stained with silver dye. Second, the cell clones with desired silver staining would be examined further by cloning the genomic PCR products into T vector using pClone007 Versatile Simple Vector Kit (TsingKe Biological Technology, Beijing, China) and inserted fragments were multiply sequenced and analyzed for base insertion and deletion (indel) mutations. After screening of 49 RIG-I, 10 MDA5 and 8 LGP2 PAM cell clones, we obtained RIG-I^-/-^, MDA5^-/-^ and LGP2^-/-^ PAM homozygous KO cell clones.

### Co-Immunoprecipitation and Western Blotting

For Co-immunoprecipitation, cells in 6-well plate (8×10^5^ cells/well) were transfected with different combinations of plasmids for 24 h, and then subjected to different treatments to activated receptor signaling. Cells were harvested and lysed in 500 μl RIPA buffer (50 mM Tris pH 7.2, 150 mM NaCl, 1% sodium deoxycholate, 1% Triton X-100) containing protease inhibitors on ice for 30 min. The cell lysates were centrifuged at high speed of 10,000g to pellet cellular debris and the cleared supernatants were transferred to new tubes. The 25-50 μl (5-10%) cleared lysates was kept as input controls and the remained were incubated with 1 μg specific antibody at 4°C overnight with shaking, further incubated with Protein A/G PLUS-Agarose (sc-2003, Santa Cruz Biotechnology) for 2-3h. Later, the agarose was thoroughly washed with RIPA and eluted with 40 μl 2×SDS sample buffer. The elution and input were both subjected to following Western-blot analysis. Protein samples containing 1×SDS sample buffer were boiled at 100°C for 5-10 min and resolved on 6-10% SDS-polyacrylamide gels. The protein bands on gels were transferred onto PVDF membranes and the membranes blocked with 5% nonfat dry milk Tris-buffered saline, pH 7.4, with 0.1% Tween-20 (TBST). Next PVDF membranes were sequentially incubated with various primary antibodies and HRP-conjugated goat anti-mouse or anti-rabbit IgG. The protein signals were detected with enhanced chemiluminescence (ECL) substrate (Tanon, China) and visualized by Western blot imaging system (Tanon, China).

### Porcine RLR Protein Purification and dsRNA Binding Assay

The HA-tagged pLGP2, pRIG-I and FLAG-tagged pLGP2, pMDA5 were transferred from pENTR4 vectors to Destination vector pDEST527 (Addgene) by LR recombination to obtain the prokaryotic expression plasmids. Then the protein expression in bacteria DE3/BL21 was induced by IPTG. The proteins in the supernatant of bacterial lysates were purified according to the instructions of Protino^®^ Ni-TED 2000 Packed Columns Kit, while the proteins in precipitation of bacterial lysates were purified according to the instructions of Micro Protein PAGE Recovery Kit. Specifically, the proteins in supernatant were loaded onto the Protino Ni-TED Packed Column for binding, then washed by LEW buffer and eluted by elution buffer according to the instructions of Protino Ni-TED 2000 Packed Columns Kit. The proteins in precipitation were separated by SDS-PAGE gel, and the target protein were cut off and put into a clean tube, then grinded into small pieces. Next, appropriate amount of solution A was added and the tube was shaked overnight at room temperature. Next day, the centrifugal supernatant was taken and the precooled solution B was added to be mixed at 4°C for 30 minutes. The supernatant was discarded after high-speed centrifugation, and the precipitated protein was dissolved with proper amount of PBS according to the instructions of Micro Protein PAGE Recovery Kit.

For dsRNA binding assay, 10 μg purified protein was mixed with 1μg dsRNA analogue poly (I:C) HMW Biotin in 500 μL PBS, incubated at 25°C for 1h with slow rotating. Then 30 μL resuspended streptavidin agarose was added and incubated at 4°C on a rotating device overnight. Next day, the protein-dsRNA-agarose was washed 3 times by centrifugation, and bound proteins eluted with 100 μl 2×SDS sample buffer by heating at 100°C for 10 min. Finally, the eluted proteins were analyzed by Western blotting.

### Confocal Fluorescence Microscopy

PAMs grown on 15 mm glass bottom cell culture dish (NEST, China) were transfected with pLGP2-HA and pRIG-I-FLAG, pLGP2-HA and pMDA5-FLAG, pRIG-I-HA and pMDA5-FLAG plasmids, respectively, using TransIT-LT1 Transfection Reagent. Twenty-four hours later, the transfected cells were infected or not with EMCV (0.01 MOI). Then the cells were fixed with 4% paraformaldehyde at RT for 30 min, and permeabilized with 0.5% Triton X-100 for 20 min. After washing with PBS, the cells were sequentially incubated with primary anti-HA rabbit pAb (1:200), anti-FLAG mouse mAb (1:200) and secondary antibodies Donkey Anti-Rabbit IgG Alexa Fluor 647 (1:500), Goat Anti-Mouse IgG H&L Alexa Fluor 594 (1:500). 293T cells grown on 15 mm glass bottom cell culture dish were transfected with pLGP2-HA and pRIG-I-FLAG, pLGP2-HA and pMDA5-FLAG plasmids, respectively, using Lipofectamine 2000. After stimulated with HMW poly I:C, fixed and permeabilized as PAMs, the cells were sequentially incubated with primary anti-HA rabbit pAb (1:200), anti-FLAG mouse mAb (1:200) and secondary antibodies Goat Anti-Rabbit IgG DyLight 488 (1:500), Goat Anti-Mouse IgG DyLight 594 (1:500). The stained cells were counter-stained with 0.5μg/ml 4’,6-diamidino-2-phenylindole (DAPI, Beyotime, China) at 37°C for 15 min to stain the cell nucleus. Lastly, both PAMs and 293T cells were visualized under laser-scanning confocal microscope (LSCM, Leica SP8, Solms, Germany) at the excitation wavelengths 488 nm, 594 nm and 647 nm, respectively.

### Promoter Driven Luciferase Reporter Gene Assays

293T cells grown in 96-well plates (3×10^4^ cells/well) were co-transfected by Lipofectamine 2000 with ISRE-firefly luciferase reporter or ELAM (NF-κB)-firefly luciferase (Fluc) reporter (10 ng/well) and β-actin *Renilla* luciferase (Rluc) reporter (0.2 ng/well), together with the indicated porcine RLR expressing plasmids or vector control (10–20 ng/well). The total DNA per well was normalized to 50 ng by adding empty pcDNA vector. PAMs grown in 96-well plates were similarly transfected using the TransIT-LT1 Transfection Reagent. About 24 hours post-transfection, the cells were subjected for stimulation or not, and then cells were harvested, lysed, and both Fluc and Rluc activities were sequentially measured using the TransDetect Double-Luciferase Reporter Assay Kit. The results were expressed as fold inductions of ISRE or ELAM (NF-κB)-Fluc compared with that of vector control after Fluc normalization by corresponding Rluc.

### RT-PCR and Quantitative RT-PCR

293T, PAMs, PK-15, IPEC-J2 or Marc-145 cells grown in 24-well plates (3×10^5^ cells) were subjected to different treatments. The treated cells were harvested and RNA extracted with TRIpure Reagent. The extracted RNA was reverse transcribed into cDNA with HiScript^®^ 1st Strand cDNA Synthesis Kit, then the target gene expressions were measured by PCR or quantitative PCR (qPCR) with ChamQ Universal SYBR qPCR Master Mix using StepOne Plus equipment (Applied Biosystems). The PCR program is denaturation at 95°C for 30 s followed by 30 cycles of 95°C for 30 s, 55°C for 30 s and 72°C for 30 s, whereas the qPCR program is denaturation at 95°C for 30 s followed by 40 cycles of 95°C for 10 s and 60°C for 30 s. The PCR primers for hIFNβ, hISG56, hISG60, hIL8, hRPL32, pIFNβ, pISG56, pISG60, pIL1β, pIL8, pTNFα, pRIG-I, pMDA5, pLGP2, pTLR3, pβ-actin, MIFNβ, MIL-1β, MIL-8, MTNF-α, Mβ-actin, swIAV-HA, swIAV-M, HSV-1 (gB), VSV (Glycoprotein), EMCV (Polyprotein), PEDV (M), PRRSV (N) and SeV (GFP) are shown in **Supplementary Table 3**. The conventional PCR products were analyzed by agarose gel electrophoresis and visualized by imaging, whereas in qPCR, the transcriptional levels of target genes were quantified using the ΔΔC_T_ calculation method.

### Virus TCID50 and Plaque Assays

Vero and Marc-145 cells in 96-well plates (5×10^4^ cells) were grown at 90% confluency, then infected with 0.1 mL cell supernatants from PEDV infected IPEC-J2 and PRRSV infected Marc-145 cells, respectively. The cell supernatants were tenfold serially diluted in 2% FBS DMEM medium before infections of Vero and Marc-145 cells, and each dilution had four to six replicates. The cytopathic effects (CPEs) were observed daily for 4-6 days, and TCID50 was calculated using the Reed-Muench method.

Vero and Marc-145 cells in 24-well plates (3×10^5^ cells) or 12-well plates (8×10^5^ cells) were grown into monolayer, then infected for 2 h with the tenfold serially diluted cell supernatants (0.2 mL for 24-well plate and 0.4 mL for 12-well plate) from virus infected cells. The infected cells were washed and overlaid with an appropriate volume of immobilizing medium of 1:1 mixture of warmed 2× DMEM with 4% FBS and a stock solution of heated 1.6% low melting agarose. Plague formation could take 1-6 days depending on the viruses being analyzed. The immobilizing medium were discarded by tipping and cells were fixed and stained with crystal violet cell colony staining solution (0.05% w/v crystal violet, 1% formaldehyde, 1×PBS and 1% methanol) for 1h at room temperature. After staining, cells were washed with tap water until the clear plaques appeared. The plaques were counted and photos were taken.

### Statistical Analysis

All the experiments are representative of three similar experiments and the representative experimental data in graphs were shown as the mean ± SD of triplicate wells. The statistical analysis was performed with *t*-test or one-way *ANOVA* where appropriate which are built within the software GraphPad Prism 6.0 (San Diego, USA).

## Results

### Characterization of Porcine RIG-I, MDA5 and LGP2 Signaling Activity

**Figure 1:**
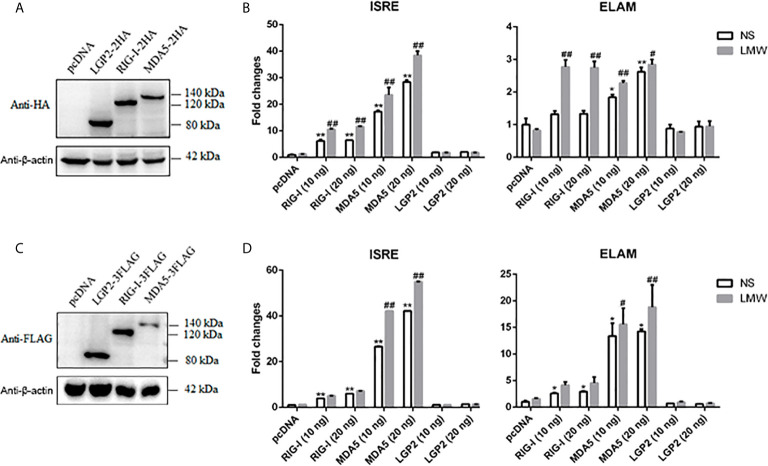
The expression of porcine LGP2, RIG-I, MDA5 and their activity in promoter assays. **(A)** The pcDNA-pLGP2-HA, pcDNA-pRIG-I-HA, pcDNA-pMDA5-HA and vector pcDNA (0.5 μg each) were transfected into 293T cells in 24-well plate (3×10^5^ cells/well) for 24 h using Lipofectamine 2000. The cells samples were detected by Western-blotting with anti-HA mAb. **(B)** 293T cells grown in 96-well plates (1×10^4^ cells/well) were transfected with 10 ng or 20 ng pcDNA-pLGP2-HA, pcDNA-pRIG-I-HA, pcDNA-pMDA5-HA, plus ISRE-Fluc/ELAM-Fluc (10 ng) and Rluc (0.2 ng), which were normalized to 50 ng/well by vector pcDNA. Twenty-four hours post transfection, the cells were transfected or not with LMW poly I:C (1 μg/ml) for 8 h. The luciferase activities were measured with Double-Luciferase Reporter Assay. **(C)** The pcDNA-pLGP2-FLAG, pcDNA-pRIG-I-FLAG, pcDNA-pMDA5-FLAG and pcDNA were transfected as in **(A)** and detected with anti-FLAG mAb. **(D)** Promoter activation by pcDNA-pLGP2-FLAG, pcDNA-pRIG-I-FLAG, pcDNA-pMDA5-FLAG were detected as in **(B)**. *p < 0.05, **p < 0.01 vs pcDNA controls. ^#^ *p* < 0.05, ^##^ *p* < 0.01 vs non-stimulated (NS) controls.

We isolated and cloned porcine RLRs RIG-I, MDA5 and LGP2, which were deposited into GenBank with the accession numbers MF358966, MF358967 and MF358968, respectively. We confirmed that these porcine RLRs are all IFN stimulated genes (ISG), upregulated by IFN and virus stimulation (**Supplementary Figure 1A**). The desired HA or FLAG tagged protein expressions of these porcine RLR members were verified by Western blotting (**Figures 1A, C**). In terms of signaling activity, we found that both porcine RIG-I and MDA5 exhibited constitutive signaling activity in ISRE and NF-κB promoter assay, with MDA5 harboring higher constitutive activity (**Figures 1B, D**). Upon stimulation by the transfection of double stranded RNA (dsRNA) analogue polyinosinic:polycytidylic acid (poly I:C-LMW), the activities of RIG-I and MDA5 were further heightened (**Figures 1B, D**). In contrast, LGP2 did not show any signaling activity with or without stimulation in ISRE and NF-κB promoter assay (**Figures 1B, D**).

**Figure 2:**
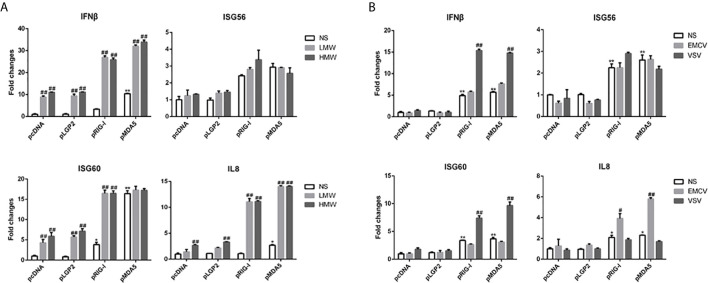
The signal activity of pLGP2, pRIG-I and pMDA5 before and after poly I:C transfection and virus infection. **(A)** 293T cells grown in 24-well plates (3×10^5^ cells/well) were transfected with HA tagged pLGP2, pRIG-I, pMDA5 or pcDNA (0.5 μg each) using Lipofectamine 2000. Twenty-four hours post transfection, the cells were transfected with LMW poly I:C or HMW poly I:C (1 μg/ml) for 8h stimulation. The harvested cells were analyzed by RT-qPCR for downstream gene expressions as indicated. **(B)** 293T cells in 24-well plates (3×10^5^ cells/well) were transfected as in **(A)** and infected with 0.01 MOI EMCV or VSV for 8 h, and analyzed by RT-qPCR. *p < 0.05, **p < 0.01 vs pcDNA controls. ^#^ *p* < 0.05, ^##^ *p* < 0.01 vs NS controls.

Next, we sought to examine the ligand recognition of dsRNA and viruses by porcine RIG-I and MDA5. In this respect, we chose two types of synthetic analog of viral dsRNA: LMW poly I:C and HMW poly I:C with the length of 0.1-0.6 kb and 1.5-10+ kb, respectively (**Supplementary Figure 1B**), as well as two viruses vesicular stomatitis virus (VSV) and encephalomyocarditis virus (EMCV). In general, short dsRNA (< 300 bp) was preferentially recognized by human/mice RIG-I whereas long dsRNA (> 1kb) was mainly recognized by human/mice MDA5 (34–36). Although LMW poly I:C is not as a commonly used agonist for RIG-I as the 5’-ppp dsRNA, it was confirmed as an effective RIG-I stimulator in several studies including our previous study (37), whereas HMW poly I:C is well known as a agonist for MDA5. In terms of virus recognitions, VSV and EMCV were recognized by RIG-I and MDA5, respectively (34, 37–39). We first examined the recognition pattern of these poly I:C and viruses by human RIG-I and MDA5 in transfected cells, the results showed as previously reported (**Supplementary Figure 1C**). In contrast, as shown in **Figure 2**, the poly I:C (LMW or HMW) activated the porcine RIG-I and MDA5 mediated downstream gene transcriptions in a similar trend (**Figure 2A** and **Supplementary Figure 1D**), and as did the two viral stimulations (**Figure 2B** and **Supplementary Figure 1D**). These results might implicate that porcine RIG-I and MDA5 do not bear the feature of preferential recognition of dsRNA and viruses, instead, both have similar and redundant ligand recognition patterns.

### The Porcine LGP2 Positively Regulates RIG-I and MDA5 Signaling Activity

**Figure 3:**
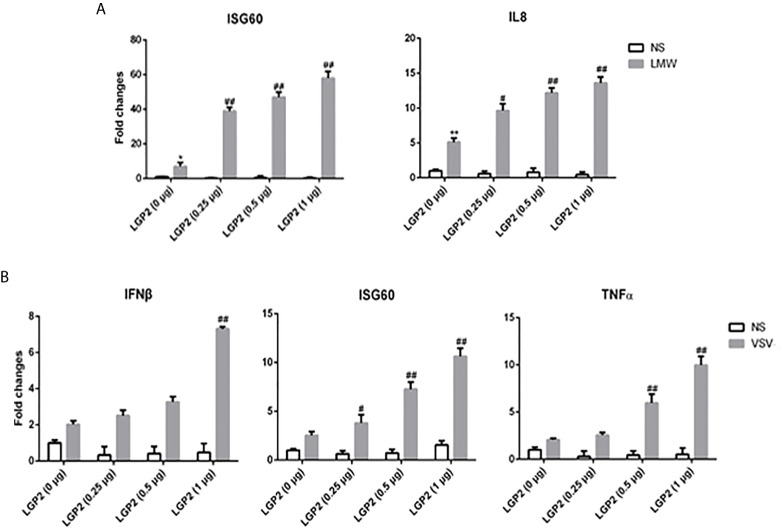
LGP2 promoted the expression of downstream genes of RIG-I and MDA5 in PAMs after LMW poly I:C transfection and VSV infection. **(A)** PAMs grown in 24-well plates (3×10^5^ cells/well) were transfected with LGP2 in different amounts (0, 0.25, 0.5, 1 μg) using Lipofectamine 3000. Twenty-four hours post transfection, the cells were stimulated with LMW poly I:C transfection (1 μg/ml) for 8h. Then the cells were analyzed by RT-qPCR for downstream gene expressions as indicated. **(B)** PAMs grown in 24-well plates were transfected as in **(A)**. The cells were infected with 0.01 MOI VSV for 8h and analyzed by RT-qPCR. *p < 0.05, **p < 0.01 vs mock transfection and NS controls. ^#^ *p* < 0.05, ^##^ *p* < 0.01 vs mock transfection and stimulated controls.

The relationship of LGP2 with RIG-I and MDA5 has been elusive (8, 9). Therefore, we embarked to explore the regulation of porcine RIG-I and MDA5 by LGP2. First, PAMs were transfected with different concentrations of porcine LGP2, and stimulated with LMW poly I:C and VSV, the downstream gene transcriptions were detected (**Figures 3A, B**). In both cases, the positive regulation of porcine RIG-I and MDA5 mediated downstream gene transcriptions by LGP2 was observed in a dose dependent manner.

**Figure 4:**
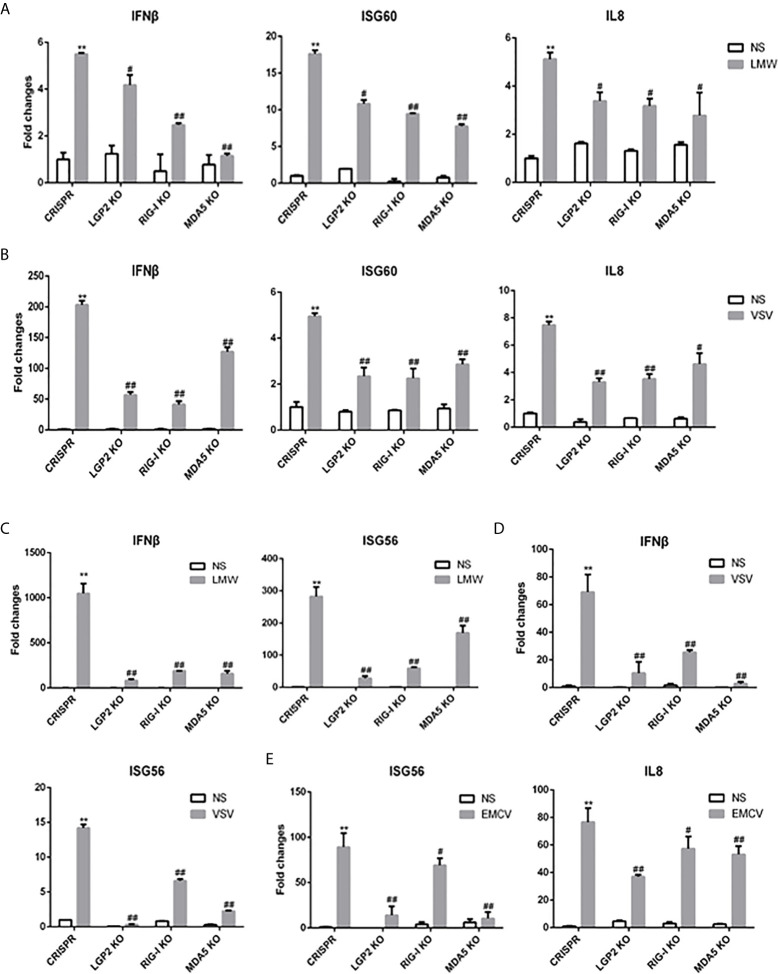
Expressions of downstream genes in CRISPR KO stable cell lines upon stimulations. **(A)** The LGP2, RIG-I and MDA5 stable KO PAMs plus CRISPR control PAMs grown in 24-well plates (3×10^5^ cells/well) were transfected with LMW poly I:C (1 μg/ml). Eight hours post transfection, the cells were analyzed by RT-qPCR for downstream gene expressions. **(B)** The LGP2, RIG-I, MDA5 stable KO and control PAMs were infected with VSV at MOI of 0.01 for 8h and analyzed as in **(A)**. **(C)** The LGP2, RIG-I and MDA5 KO PK15 cells plus CRISPR control PK15 cells grown in 24-well plates (3×10^5^ cells/well) were transfected and analyzed as in **(A)**. **(D, E)** The LGP2, RIG-I, MDA5 stable KO PK15 cells were infected with VSV **(D)** or EMCV **(E)** at MOI of 0.01 and analyzed as in **(A)**. **p < 0.01 vs CRISPR NS controls. ^#^ *p* < 0.05, ^##^ *p* < 0.01 vs CRISPR stimulated controls.

Second, to further examine the regulation of porcine LGP2 in RIG-I/MDA5 mediated signaling, the CRISPR/Cas9 method was used to knockout endogenous porcine LGP2, RIG-I and MDA5, respectively. The gRNAs designed for LGP2, RIG-I and MDA5 were tested for efficacy and specificity (**Supplementary Figures 2A–C**) and the effective lentiviral gRNAs were used for lentivirus package and preparation of the stable KO PAMs and PK15 cells. As shown in **Figure 4**, the LMW poly I:C and VSV induced downstream IFNβ, ISG60 and IL8 gene transcription in LGP2 KO PAMs were all decreased relative to CRISPR control PAMs (**Figures 4A, B**) and similar results were obtained for IFNβ and ISG56 gene transcription in stable LGP2 KO PK15 cells (**Figures 4C, D**). Also, EMCV induced ISG56 and IL8 gene transcriptions were decreased in LGP2 KO PK15 cells (**Figure 4E**). There results clearly suggested that porcine LGP2 positively regulates the collective signaling activity of RIG-I and MDA5. Additionally, in RIG-I KO and MDA5 KO cells, the LMW poly I:C, VSV and EMCV induced downstream gene transcriptions were all decreased between both types of KO cells. Together with the results showing that ectopic RIG-I and MDA5 respond similarly to distinct RNAs or viruses, our results also suggested that porcine RIG-I and MDA5 play a similar and cooperative role in recognition of different RNAs and viruses.

**Figure 5:**
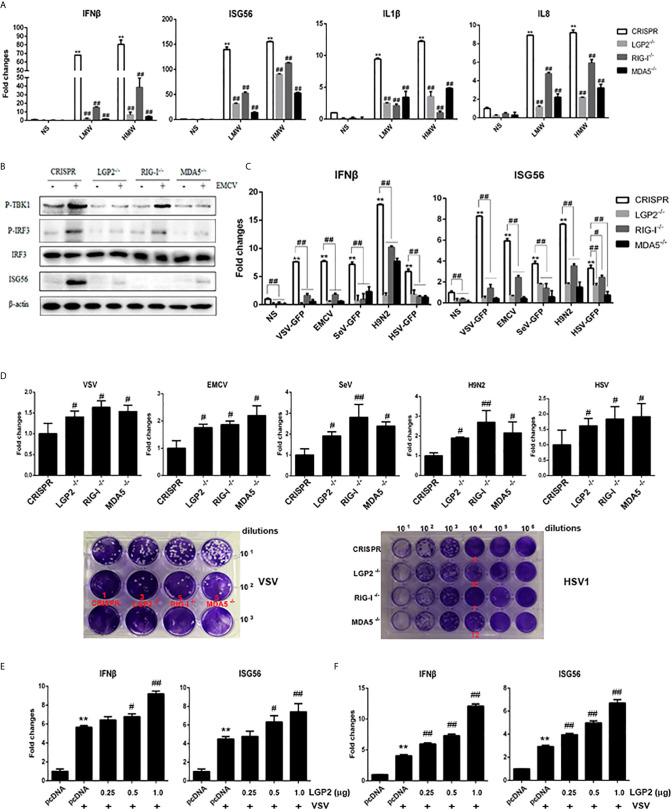
Expressions of cellular genes and viral replications in LGP2, RIG-I and MDA5 homozygous KO PAM cell clones. **(A)** The LGP2^-/-^, RIG-I^-/-^ and MDA5^-/-^ PAMs plus CRISPR control PAMs grown in 12-well plates (5×10^5^ cells/well) were transfected with LMW poly I:C or HMW poly I:C (1 μg/ml) for 8h. The expression of downstream cellular genes were analyzed by RT-qPCR. **(B)** The LGP2^-/-^, RIG-I^-/-^ and MDA5^-/-^ PAMs plus CRISPR control PAMs grown in 12-well plates (5×10^5^ cells/well) were infected with EMCV at 0.01 MOI for 8 h. The cells were detected by Western-blotting with the indicated antibodies. **(C, D)** The LGP2^-/-^, RIG-I^-/-^ and MDA5^-/-^ PAMs plus CRISPR control PAMs in 12-well plates (5×10^5^ cells/well) were infected with various viruses at MOI of 0.01 for 8-12 h and analyzed by RT-qPCR for the expressions of downstream cellular genes **(C)** and viral genes (up panel, **D**). The supernatants from VSV and HSV1 infected PAMs were subjected to infection of Vero cells and the viral plaques were visualized at 30 h and 72 h post infection, respectively. The countable plaque numbers are labeled in red color below the wells of cell plates (low panel, **D**). **(E, F)** MDA5^-/-^ PAMs **(E)** and RIG-I^-/-^ PAMs **(F)** in 24-well plates were transfected different amounts of LGP2 and stimulated with 0.01 MOI VSV for 8 h. The downstream IFNβ and ISG56 were analyzed by RT-qPCR. **p < 0.01 vs NS controls. ^#^ *p* < 0.05, ^##^ *p* < 0.01 vs CRISPR controls in **(A–D)**, or pcDNA VSV controls in **(E, F)**.

To ensure the roles of endogenous porcine LGP2, RIG-I and MDA5 in the signaling activity, we further generated the porcine LGP2, RIG-I and MDA5 homozygous KO PAM cell clones (**Supplementary Figures 2D–F**). Upon stimulations of either HMW or LMW poly I:C, the IFNβ, ISG56, IL1β and IL8 gene transcriptions were all impaired in LGP2^-/-^, RIG^-/-^ and MDA5^-/-^ PAMs relative to CRISPR control PAMs (**Figure 5A**). The EMCV activated TBK1 and IRF3 phosphorylation and downstream ISG56 production were also decreased in LGP2^-/-^, RIG^-/-^ and MDA5^-/-^ PAMs relative to CRISPR control PAMs (**Figure 5B**). Next we stimulated the PAM cell clones with a panel of viruses including VSV, Sendai virus (SeV), EMCV, swIAV H9N2 and Herpes simplex virus (HSV-1). All these virus infection induced gene transcriptions of IFNβ, ISG56, TNFα, IL1β and IL8 were reduced in in LGP2^-/-^, RIG^-/-^ and MDA5^-/-^ PAMs compared with control PAMs (**Figure 5C** and **Supplementary Figure 3A**). Simultaneously, the virus replications of VSV, SeV, EMCV, H9N2 and HSV-1 were significantly upregulated in LGP2^-/-^, RIG^-/-^ and MDA5^-/-^ PAMs (**Figure 5D** and **Supplementary Figure 3B**). Generally, the reductions of kinase phosphorylations, downstream gene transcriptions and protein production, and the upregulations of virus replications were relatively comparable in three types of KO PAM cell clones, which again implicates a cooperative relationship between three porcine RLR members. To dissect the regulatory role of LGP2 in RIG-I or MDA5 signaling alone, we used the MDA5^-/-^ PAM (with RIG-I) and RIG^-/-^ PAM (with MDA5) transfected with different amounts of LGP2, and found that in both cases, LGP2 enhanced the VSV activated downstream IFNβ and ISG56 gene transcriptions in dose dependent manners (**Figures 5E, F**). It suggested that porcine LGP2 not only positively regulates MDA5, but also positively regulates RIG-I.

**Figure 6:**
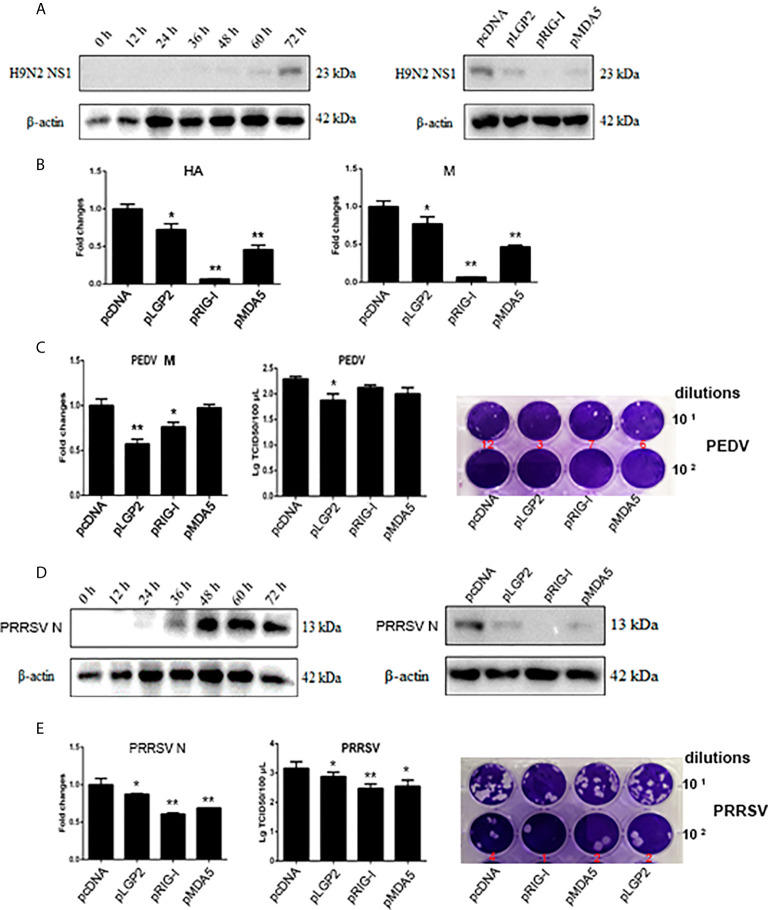
The antiviral effects of ectopic porcine LGP2, RIG-I and MDA5. **(A, B)** PAMs were infected with swine influenza virus H9N2 at MOI of 0.01 for different hours and the viral protein NS1 was detected by Western-blotting with anti-NS1 mAb (left panel, **A**). PAMs grown in 12-well plates (5×10^5^ cells/well) were transfected with LGP2, RIG-I and MDA5 (1 μg each) using TransIT-LT1 Transfection Reagent. The cells were infected with 0.01 MOI H9N2 for further 63 h and analyzed by Western-blotting for NS1 protein expression (right panel, **A**) and RT-qPCR for viral HA and M gene expressions **(B)**. **(C)** IPEC-J2 cells grown in 12-well plates (5×10^5^ cells/well) were transfected as in **(A)** and infected with 0.01 MOI PEDV (YC2014 strain) for 24 h. The cells were analyzed by RT-qPCR for viral M gene expression (left). The cell supernatants were examined by TCID50 assay (middle) and plaque assay (right), which were performed with Vero cells for 6 day infection and the countable plaque numbers are labeled in red color below the wells of plate. **(D, E)** Marc-145 cells were infected with 0.01 MOI PRRSV (JXA1-R strain) for different hours and the virus protein N was detected by Western-blotting with anti-N mAb (left panel, **D**). Marc-145 cells grown in 12-well plates (5×10^5^ cells/well) were transfected as in **(A)**, and the cells were infected with 0.01 MOI JXA1-R for 36h and analyzed by Western-blotting for N protein expression (right panel, **D**) and RT-qPCR for viral N gene (left, **E**). The cell supernatants were examined by TCID50 assay (middle, **E**) and plaque assay (right, **E**), which were performed with Marc-145 cells for 6 day infection and the countable plaque numbers are labeled in red color below the wells of plate *p < 0.05, **p < 0.01 vs pcDNA controls.

To further assess the positive regulation role of LGP2, we examined if porcine LGP2 exerts antiviral function in transfected cells against several porcine viruses including swIAV H9N2, PEDV and PRRSV. In all three cases, similar to transfected porcine RIG-I or MDA5, transfected porcine LGP2 suppressed viral replications (**Figure 6**). Specifically, LGP2 in PAMs was shown to suppress H9N2 NS1 protein expression (**Figure 6A**), and HA and M gene transcriptions (**Figure 6B**) at 63 h post infection in WB and RT-qPCR, respectively (**Figures 6A, B**). LGP2 in transfected IPEC-J2 cells inhibited PEDV M gene transcription in RT-qPCR and lowered the PEDV titers in TICD50 and plaque assays (**Figure 6C**). LGP2 in Marc-145 cells inhibited both PRRSV N protein expression (**Figure 6D**) and gene transcription (left, **Figure 6E**) at 36 h post infection. In addition, LGP2 reduced the PRRSV titers in TCID50 and plaque assays (middle and right, **Figure 6E**).

### Porcine LGP2 Interacts With RIG-I and MDA5 Upon Activation and Promotes Their RNA Binding

**Figure 7:**
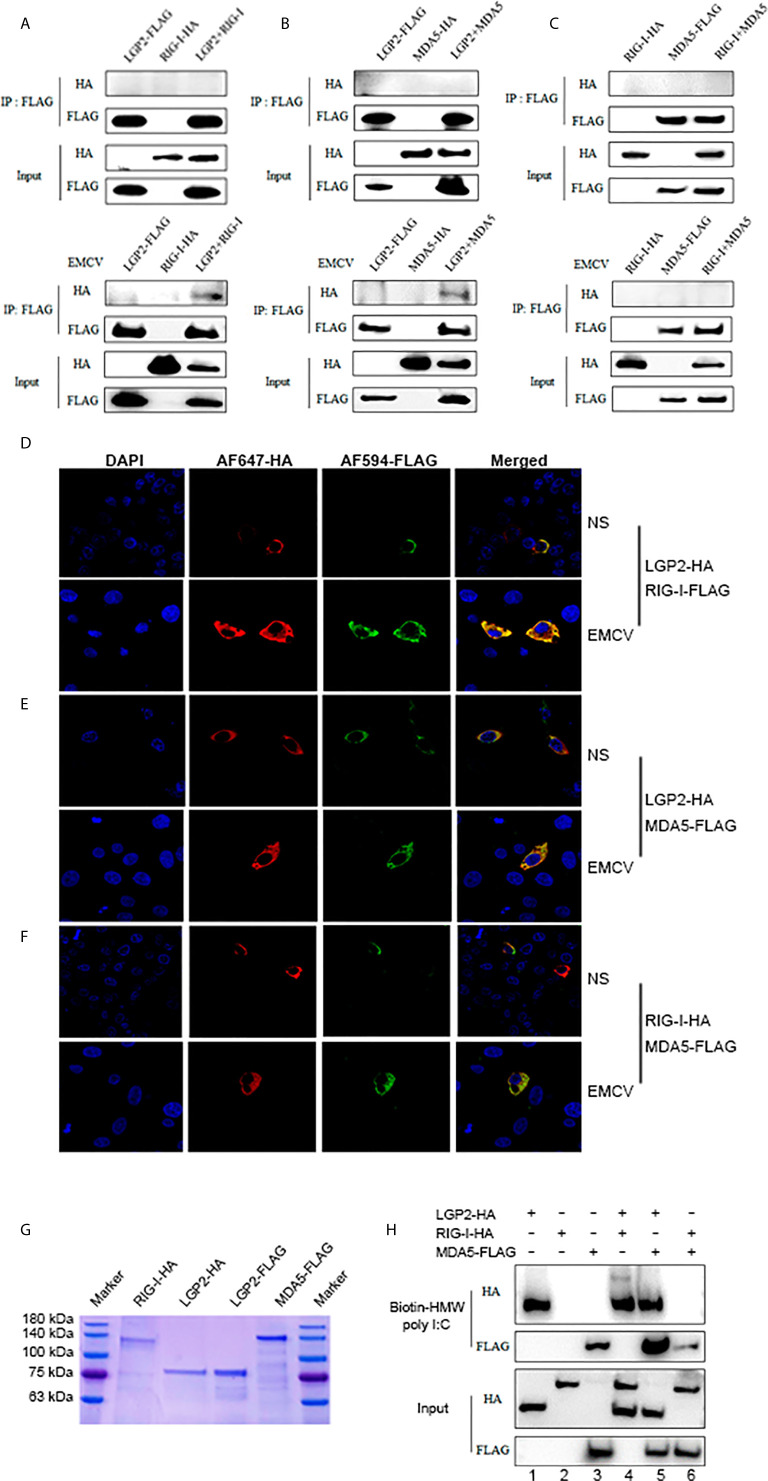
Porcine LGP2 interacts RIG-I and MDA5 upon activation and promotes their RNA binding. **(A)** pLGP2-FLAG (1 μg) and pRIG-I-HA (1.5 μg) were co-transfected into 293T cells in 6-well plate (8×10^5^ cells/well) for 24 h, then infected with or without 0.01 MOI EMCV for 8 h. The cells lysates were immunoprecipitated with anti-FLAG mAb and subjected to Western-blotting using the indicated antibodies. **(B)** pLGP2-FLAG and pMDA5-HA were co-transfected into 293T, and the transfected cells were treated and processed as in **(A)**. **(C)** pMDA5-FLAG and pRIG-I-HA were co-transfected into 293T cells, and the transfected cells were treated and processed as in **(A)**. **(D)** PAMs grown on 15-mm glass bottom cell culture dish (5×10^5^ cells) were co-transfected with pLGP2-HA (0.75 μg) and pRIG-I-FLAG (0.75 μg) for 24 h, then infected with or without EMCV for 8 h. The cells were examined for co-localization by con-focal fluorescence microscopy. **(E)** pLGP2-HA and pMDA5-FLAG were co-transfected and the transfected PAMs were treated and examined for co-localization as in **(D)**. **(F)** pRIG-HA and pMDA5-FLAG were co-transfected, and transfected PAMs were treated and examined for co-localization as in **(D)**. **(G)** The purified pLGP2-HA, pLGP2-FLAG, pRIG-HA and pMDA5-FLAG were analyzed by SDS-PAGE. **(H)** pLGP2-HA, pRIG-I-HA and pMDA5-FLAG were incubated in tubes alone or together as indicated, then 1μg dsRNA poly (I:C) HMW Biotin and 30 μL streptavidin agarose were added for binding. The dsRNA bound proteins were analyzed by Western-blotting.

We speculated that porcine LGP2 may positively regulate RIG-I and MDA5 *via* several ways: interacting directly with RIG-I and MDA5 to promote their activation; promoting the ligand RNA binding by RIG-I and MDA5; helping the interactions between RIG/MDA5 and the downstream common adaptor MAVS. The interactions between porcine LGP2, RIG-I and MDA5 in transfected cells were first detected by Co-immunoprecipitation. Without cell activation, no any interaction between three porcine RLRs could be observed (upper panels, **Figures 7A–C** and **Supplementary Figure 4**). With EMCV infection, the interactions of LGP2 and RIG-I, as well as LGP2 and MDA5 appeared, but no interaction between RIG-I and MDA5 occurred which are consistent with previously observed (13) (lower panels, **Figures 7A–C**). Accordingly, the cytosolic co-localizations of LGP2 and RIG-I, as well as LGP2 and MDA5 were obvious in transfected PAMs and 293T cells after cell stimulation by EMCV and HMW poly I:C (**Figures 7D–F** and **Supplementary Figure 5**). The interactions between the three porcine RLRs and the adaptor MAVS in the presence or absence of EMCV infection were also examined, but significant interaction could not be detected (**Supplementary Figure 4**).

We further examined the RNA binding of porcine LGP2, RIG-I and MDA5 using the HMW poly I:C pull-down assay. The RNA pull-down assay was performed with purified porcine LGP2, RIG-I and MDA5 proteins (**Figure 7G**). HMW poly I:C could successfully pull down LGP2 and MDA5 (Lanes 1, 3, **Figure 7H**), which is consistent with previous knowledge that both LGP2 and MDA5 bind similarly the blunt-ended dsRNA such as poly I:C (11, 40). However, HMW poly I:C failed to pull down porcine RIG-I (Lanes 2, **Figure 7H**) even though previous studies showed that RIG-I could bind to RNA with higher affinity than MDA5 (17, 41). We reasoned that HMW poly I:C, due to its long length, is not the optimal ligand for RIG-I. In the presence of porcine LGP2, the RNA binding of MDA5 was definitely enhanced (Line 5 versus line 3, **Figure 7H**). Similarly, in the presence of porcine LGP2, the RNA bound RIG-I appeared (Line 4 versus line 2, **Figure 7H**), suggesting an enhancement of the RNA binding of RIG-I by LGP2. In addition, in the presence of porcine RIG-I, the RNA bound MDA5 was subjected to a slight decrease (Line 6 versus line 3, **Figure 7H**).

### Porcine LGP2 ATPase Sites Are Not Required for Its Positive Regulation of RIG-I and MDA5

**Figure 8:**
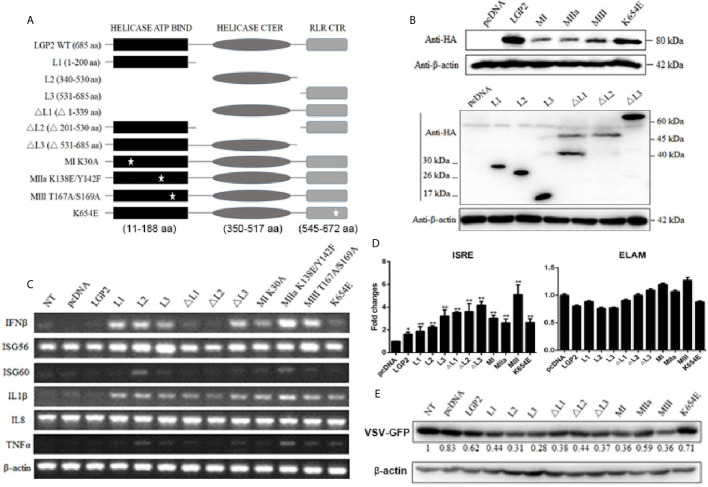
Expression of pLGP2 mutants and their positive regulation on pRIG-I and pMDA5. **(A)** The pLGP2 protein structure and domains predicted by UniProt (https://www.uniprot.org). **(B)** The protein expressions of pLGP2 domains, deletion mutants and point mutants in transfected 293T cells. **(C)** PAMs grown in 12-well plates (5×10^5^ cells) were transfected with pLGP2 mutants (1 μg each) using TransIT-LT1 Transfection Reagent for 24 h. The cells were analyzed by RT-PCR for the expressions of downstream genes. **(D)** PAMs grown in 96-well plates (1×10^4^ cells/well) were transfected with pLGP2 and its mutants (20 ng each) plus ISRE-Fluc/ELAM-Fluc (10 ng) and Rluc (0.2 ng), using the TransIT-LT1 Transfection Reagent. Twenty-four hours post transfection, the luciferase activities were measured. **(E)** PAMs grown in 12-well plates (5×10^5^ cells) were transfected as in **(C)**, then infected with 0.01 MOI VSV-GFP for 12 h. The cells were analyzed by Western-blotting with anti-GFP mAb, and the GFP densitometric values after normalization by actin were shown below. *p < 0.05, **p < 0.01 vs pcDNA controls.

LGP2 comprises three domains: N-terminal helicase ATP binding domain (ATP bind), helicase C-terminal domain (CTER) and C-terminal regulatory domain (CTR) with the conserved helicase motifs I, II and III in the helicase ATP binding domain. These motifs I, II, III are implicated in ATPase hydrolysis activity of LGP2, whereas the K654 site in CTR is involved in dsRNA binding (42, 43). To dissect the roles of individual domains of porcine LGP2 in the regulation of RIG-I and MDA5 signaling, the each LGP2 domains and domain deletion mutants were produced (**Figure 8A**). To analyze the roles of ATPase hydrolysis and RNA binding of porcine LGP2 in the regulation, the helicase motif mutants MI (K30A), MIIa (K138E/Y142F), MIII (T167A/S169A) and RNA binding mutant K654E were prepared (**Figure 8A**). The correct expressions of these LGP2 mutant proteins were confirmed by Western-blotting (**Figure 8B**). Surprisingly, when these porcine LGP mutants were transfected into PAMs, the downstream IFNβ, ISG56, ISG60, IL1β and TNFα transcriptions were significantly activated (**Figure 8C**). All the LGP2 mutants also exhibited ISRE activity in promoter assay but no NF-κB activity appeared (**Figure 8D**). Furthermore, after these transfected PAMs were infected with VSV, corresponding to the inductions of downstream IFNβ, ISGs and proinflammatory cytokines, most of the LGP2 mutants showed anti-VSV activity, which was even greater than wild type LGP2 (**Figure 8E**).

**Figure 9:**
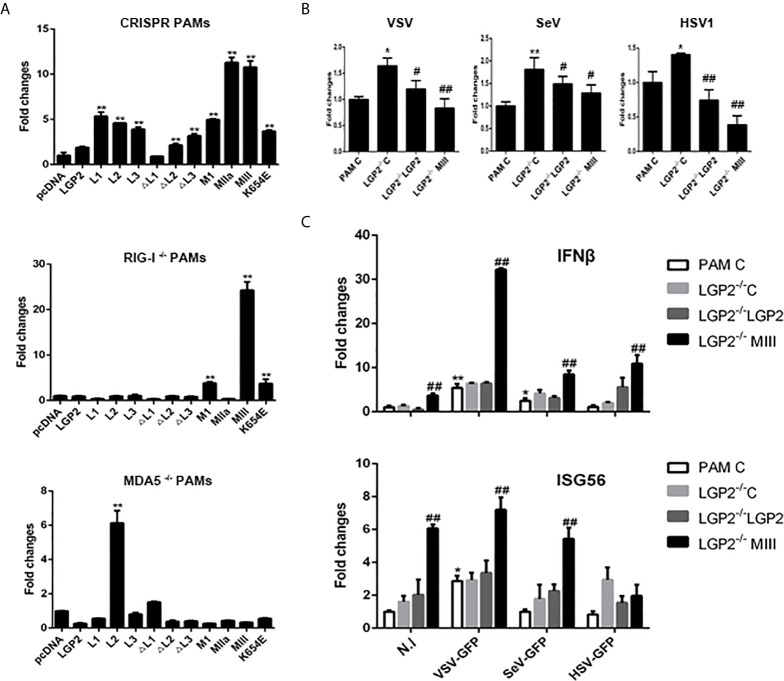
The porcine LGP2 mutant activity in RIG^-/-^ and MDA5^-/-^ PAMs and the antiviral activity. **(A)** RIG^-/-^, MDA5^-/-^ and CRISPR control PAMs in 12-well plates (5×10^5^ cells) were transfected with pLGP2 and its mutants (0.75 μg each). Twenty-four hours later, the cells were analyzed by RT-qPCR for the downstream IFNβ gene transcriptions. **p < 0.01 vs pcDNA controls. **(B, C)** LGP2^-/-^ PAMs were transfected with 0.75 μg pLGP2, its constitutive MIII mutant or pcDNA control **(C)** for 24 h. PAMs transfected with pcDNA was used as a control (PAM C). The transfected cells were infected with 0.01 MOI VSV, SeV and HSV-1 for 8 h, and analyzed by RT-qPCR for virus replications **(B)**, as well as for cellular downstream IFNβ and ISG56 expressions **(C)**. *p < 0.05, **p < 0.01 vs PAM C or N. I ^#^ *p* < 0.05, ^##^ *p* < 0.01 vs LGP2^-/-^ PAM C.

Since LGP2 itself is signaling inactive to IFN, we wondered if the constitutive activity of LGP2 mutants was coming from its regulating receptors RIG-I and MDA5. Therefore, the LGP2 mutants were transfected into RIG-I^-/-^ and MDA5^-/-^ PAMs, and downstream gene transcriptions were analyzed. As shown in **Figure 9A**, relative to control PAMs, the constitutive signaling activity to induce downstream IFNβ gene transcription by LGP2 mutants in RIG^-/-^ and MDA5^-/-^ PAMs were both dampened. Nevertheless, the constitutive activity of LGP2 mutants MI, MIII and K654E in RIG^-/-^ PAMs and LGP2 L2 domain in MDA5^-/-^ PAMs still remained, suggesting certain level of redundancy between porcine RIG-I and MDA5. To examine the antiviral activity of porcine LGP2 and its mutants, LGP2 and the constitutively active MIII mutant (T167A/S169A) were expressed in LGP2^-/-^ PAMs, and the anti-VSV, anti-SeV and anti-HSV-1 activity measured (**Figure 9B**). As expected, VSV, SeV and HSV-1 replications in LGP2^-/-^ PAM were upregulated, whereas the reconstitution of LGP2 expression suppressed all the viral replications, and reconstitution with active LGP2 MIII mutant further reduced the viral replications (**Figure 9B**). Accordingly, the upregulations of downstream gene transcriptions of IFNβ and ISG56 by active LGP2 MIII mutant were observed in the absence as well as in the presence of virus infections (**Figure 9C**).

## Discussion

Human/mice RLRs RIG-I and MDA5 utilize distinct and similar activation mechanism. Under steady state, RIG-I CARDs is bound and auto-inhibited by the Hel-2i region of the helicase. Upon binding of RIG-I C-terminal RD to RNA ends, the conformation of RIG-I changes so that the RNA further binds to Hel-2i, and thus the auto-inhibition of CARDs is lifted (20). Further, through K63-ubiquitination/polyubiquitin binding of the CARDs and/or the filament formation by RIG-I RD-helicase along the dsRNA chain, the CARDs are tetramerized into signaling effective stable lock-washer structure (7, 44). In the absence of RNA, MDA5 adopts an open and flexible structure, and thus ectopic MDA5 exhibits higher constitutive activity, which was also observed for porcine MDA5 (**Figures 1B, D**). Nevertheless, MDA5 has very similar activation mechanism where the RD-helicase binds with dsRNA and conformational change exposes the N terminal CARDs, which form tetramer structure. Because of the long dsRNA recognized by MDA5, the CARDs tetramer is stabilized mainly through the filament formation along the dsRNA by RD-helicase, and thus less dependent on K63-polyubiquitin (7, 44). In both cases, the effective CARDs tetramer will serve as a platform and nucleate downstream adaptor MASV on the mitochondrial and peroxisomal membranes, which aggregates into prion-like signaling complex (10, 44).

Despite that previous studies demonstrated a differential recognition of dsRNAs and viruses by human/mice RIG-I and MDA5 (7, 21, 34–36), we observed the similar recognitions of different dsRNAs and viruses and activations of downstream gene transcriptions by porcine RIG-I and MDA5. First, in transfected cells, porcine RIG-I and MDA5 responded similarly to two different poly I:C or two different RNA viruses (**Figure 2** and **Supplementary Figure 1D**). In parallel, we confirmed LMW poly I:C and VSV are preferred by human RIG-I, whereas HMW poly I:C and EMCV are favored by human MDA5 in transfected cells (**Supplementary Figure 1C**). Second, in porcine RIG KO and MDA5 KO stable cells and homozygous PAM cell clones, the downstream signaling, gene transcriptions and protein expression are all reduced in a similar way in response to various dsRNAs and viruses (**Figures 4**, **5**). Third, in the antiviral assay, porcine RIG-I and MDA5 exhibited comparable strengths against Influenza virus and PRRSV, two porcine RNA viruses (**Figure 6**). Not only presenting similarities, porcine RIG-I and MDA5 also showed cooperation in the recognition of RNAs and viruses since in both RIG KO and MDA5 KO cells, there were significant reductions of activity upon stimulations (**Figures 4**, **5**). Therefore, it appears that certain differences between porcine RIG-I and MDA5 and their human counterparts can be discerned.

Importantly, we found porcine LGP2 positively regulates porcine MDA5 as well as RIG-I activity. Positive regulation of MDA5 activation by physiological low concentration of LGP2 was widely accepted (15, 45, 46) and the biochemical and molecular mechanism recently became clear (17, 18, 43, 47): MDA5 binds dsRNA weakly and the filament formation on long dsRNA chain is slow and unstable. On the contrary, LGP2 binds dsRNA with the highest affinity among three RLRs, regardless of 5’-PPP and RNA length, but not forming filaments (18, 40, 48). By utilizing its ATP hydrolysis activity, LGP2 is able to not only increase initial binding of dsRNA by MDA5, but also stabilize the shorter MDA5 filaments on dsRNA which are signaling active, thus promoting MDA5 activity. Consistent with these findings and conclusions, we showed that porcine LGP2 positively regulates MDA5 (**Figures 3**–**5**), interacts with MDA5 and enhances its dsRNA binding (**Figure 7**). On the other hand, the positive regulation of RIG-I by LGP2 has not been well recognized so far with contradictory conclusions (8, 9, 15, 46, 49, 50). In our conditions using transfected cells and gene KO cells (**Figures 3**–**5**), we concluded that porcine LGP2 positively regulates RIG-I as well. Further, porcine LGP2 interaction with RIG-I and the enhanced porcine RIG-I binding to dsRNA in the presence of LGP2 provide a direct evidence for positive regulation (**Figure 7**). Additionally, we showed that porcine LGP2 serves as an broad antiviral protein against various porcine viruses including swIAV H9N2 and PR8 H1N1 which should be sensed mainly by porcine RIG-I (51) (**Figure 6** and not shown). Actually, there have been a number of studies which demonstrated the broad spectrum of anti-RNA virus functions by LGP2 from different species (52–57). Several RNA helicases including DDX3, DHX29, DHX36 and DDX60 are required for both RIG-I and MDA5 mediated IFN production after RNA virus infection (58). Likely and similarly, porcine LGP2 is involved in both RIG-I and MDA5 activation and downstream IFN and other gene transcriptions. In addition to its anti-RNA virus activity, LGP2 is also implicated in anti-DNA virus and anti-intracellular bacterial replications (59).

All RLR members are ATPase proteins and ATP hydrolysis plays regulatory roles in RLR activation. For RIG-I, ATP hydrolysis was reported to drive RIG-I translocation along 5’-PPP dsRNA chain, forming signaling active filaments (60, 61). For MDA5, ATP hydrolysis was shown to actually dissociate MDA5 from long RNA chain (62, 63), probably contributing to shorter and signaling effective filaments. For LGP2, ATP hydrolysis enables it to efficiently engage diverse dsRNA species, which promotes MDA5 recognition of dsRNA, shorter filament formation and activation (47). In this case, both ATP hydrolysis and RNA binding of LGP2 are required for positive regulation of MDA5 (18, 43). However, the roles of ATP hydrolysis in RLR activation and antiviral signaling are very complex. RIG-I, MDA5 and LGP2 all contain conserved helicase motifs I-VI in helicase domain, with all essential for ATP hydrolysis activity and some for RNA binding (42). Among the mutants, RIG-I MIII and MDA5 MI, MIII exhibited constitutive activity, suggesting that the ATP hydrolysis of RIG-I and MDA5 was not required for their activation and signaling (42). In our study, we initially sought to explore the role of ATP hydrolysis activity in the LGP2 function as a regulator, but we observed idiosyncratic phenotypes of porcine LGP2: The ATPase inactive mutants MI, MIIa and MIII exhibited obvious constitutive activity in transfected PAMs (**Figures 8**, **9**). More surprisingly, each individual domains (L1, L2, L3) and ΔL3 mutant also presented constitutive activity, whereas all the mutants including RNA binding defective mutant K654E were able to induce proinflammatory IL1β transcription (**Figure 8C**). These constitutive activities of porcine LGP2 mutants came from RIG-I and MDA5 since the activities of most mutants disappeared in RIG-I^-/-^ or MDA5^-/-^ PAMs (**Figure 9A**), implying that complexity of porcine LGP2 regulation of RIG-I and MDA5. Although we do not know the underlying mechanism regarding unique regulation of RIG-I and MDA5 by porcine LGP2 which shares 82% AA identity to human LGP2, it is warranted for further investigation.

**Figure 10:**
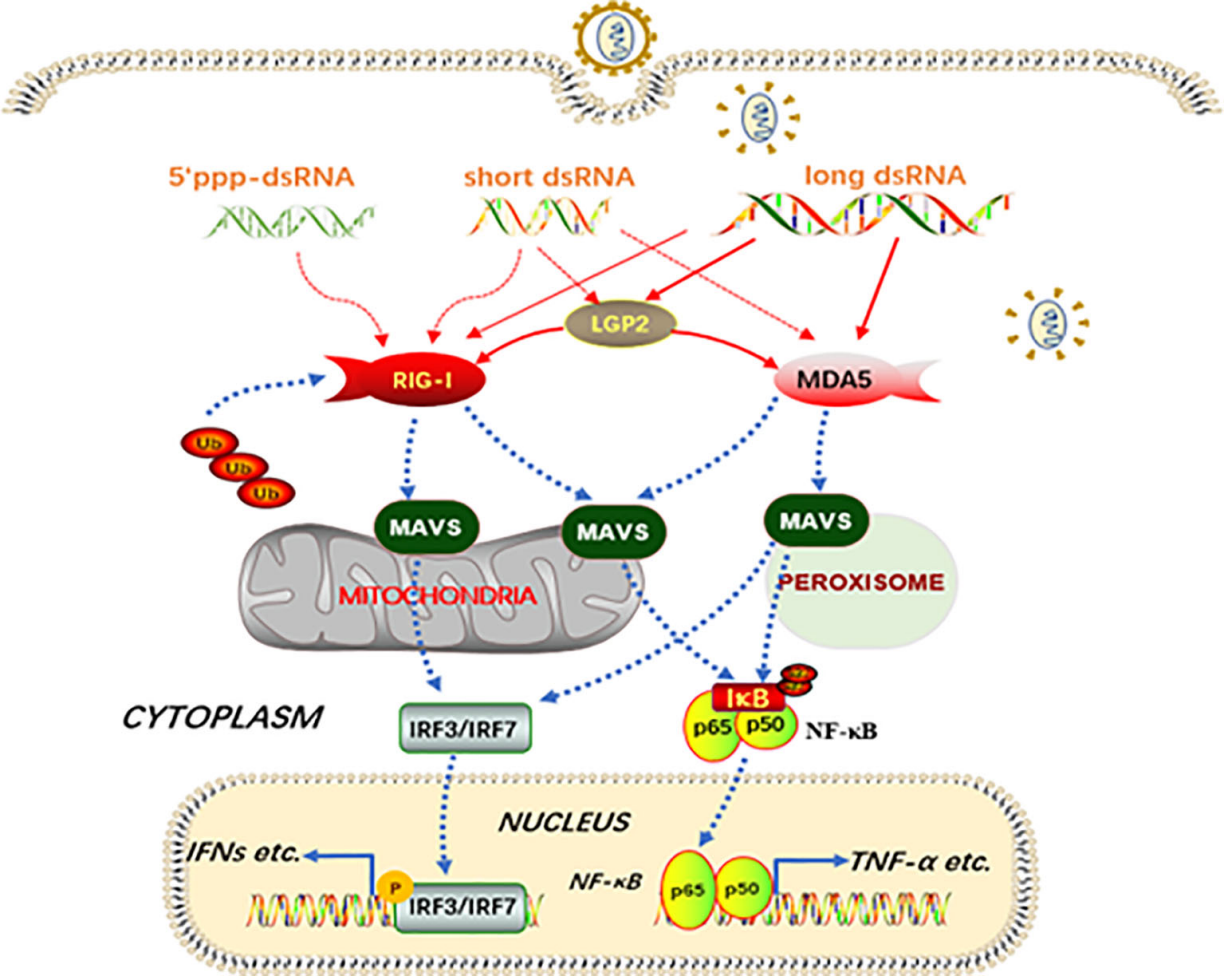
Schematic diagram of porcine RIG-I and MDA5 signaling and the regulation by LGP2. Porcine RIG-I and MDA5 similarly recognize dsRNA and sense viruses regardless of the lengths of dsRNA and the affinity of binding to dsRNA, to trigger downstream signaling and gene transcription. The porcine LGP2, binding with dsRNA in a similar way to MDA5, is able to promote the dsRNA binding by both MDA5 and RIG-I, and thus downstream signaling. Intriguingly, during the positive regulation by LGP2, the ATPase hydrolysis and dsRNA binding activity seem dispensable. The solid lines refer to confirmed interactions in this study, whereas the dashed lines are based on previous knowledge.

Collectively, in this study, we characterized signaling functions of porcine innate immune RLRs RIG-I, MDA5 and LGP2, and investigated the regulation of RIG-I and MDA5 by porcine LGP2 and the underlying mechanisms (**Figure 10**). We found that porcine RIG-I and MDA5 have similar and cooperative signaling relationship, and porcine LGP2 positively regulates RIG-I and MDA5 signaling *via* the enhancement of RNA binding by the two receptors (**Figure 10**). The ATPase activity and RNA binding of porcine LGP2 are not required for its positive regulation of RIG-I and MDA5 activity; instead porcine LGP2 exhibits complex regulation opening the doors for further study.

## Data Availability Statement

The original contributions presented in the study are included in the article/**Supplementary Material**. Further inquiries can be directed to the corresponding authors.

## Author Contributions 

JZ and NC conceived and designed the experiments. SL, JY, YZ, HW, XJ, JL, QS, YX, and XL performed the experiments; WZ, NC, FM, and JZ analyzed the data. SL, NC, and JZ wrote the paper. All authors contributed to the article and approved the submitted version.

## Funding

The work was partly supported by the National Natural Science Foundation of China (31872450; 31672523; 31802172), National Key Research and Development Program of China (2017YFD0502301), and A Project Funded by the Priority Academic Program Development of Jiangsu Higher Education Institutions (PAPD). NC is supported by High Talent Supporting Program of Yangzhou University and Natural Science Foundation of Jiangsu Province (BK20170492).

## Conflict of Interest

The authors declare that the research was conducted in the absence of any commercial or financial relationships that could be construed as a potential conflict of interest.

## Supplementary Material

Supplementary Figure 1** to **Figure 2 pRIG-I, pMDA5, and pLGP2 expressions can be induced by IFNβ and the activations of RIG-I and MDA5 by different agonists. **(A)** PAMs grown in 12-wells plates (5×10^5^ cells) were stimulated with various viruses (0.01 MOI each), LMW poly I:C transfection (1μg/ml), HMW poly I:C addition (10 μg/ml), or human IFNβ. The levels of porcine RLRs transcriptions were detected by RT-PCR. **(B)** The molecular weights of LMW and HMW poly I:C were detected by nucleic acid electrophoresis. **(C)** 293T cells grown in 24-well plates (3×10^5^ cells/well) were transfected with human RIG-I and MDA5 (0.5 μg each) using Lipofectamine 2000. Twenty-four hours post transfection, the cells were transfected with LMW poly I:C or HMW poly I:C (1 μg/ml) for 8 h (up), infected with 0.01 MOI EMCV or VSV for 8 h (down). The downstream genes were detected by RT-PCR as indicated. **(D)** 293T cells were transfected and stimulated as in **Figure 2**, and the cells were analyzed by RT-PCR for the expressions of downstream genes.

Supplementary Figure 2 **to** Figures 4, 5 The efficacy and specificity of CRISPR gRNAs and the verification of homozygous KO PAM cell clones. **(A)** The pLGP2-HA were co-transfected into 293T cells with gRNAs designed for pLGP2, pRIG-I and pMDA5. **(B)** The pRIG-I-HA were co-transfected into 293T cells with gRNAs designed for RIG-I, pMDA5 and pLGP2. **(C)** The pMDA5-HA were co-transfected into 293T cells with gRNAs designed for pMDA5, pRIG-I and pLGP2. The HA tagged pLGP2, pRIG-I and pMDA5 were analyzed by Western-blotting. **(D–F)** The genome DNA regions covering the gRNAs were amplified by PCR using the primers as shown in **Supplementary Table 2** and the PCR products of individual PAM clones of pLGP2 **(D)**, pRIG-I **(E)** and pMDA5 **(F)** were cloned into T vectors and sequenced for base insertion/deletion mutations.

Supplementary Figure 3 **to** Figures 5C, D
**(A)** Expression of downstream genes in LGP2^-/-^, RIG^-/-^ and MDA5^-/-^ PAMs infected with various viruses. The LGP2^-/-^, RIG-I^-/-^ and MDA5^-/-^ PAMs plus CRISPR control PAMs in 12-well plates (5×10^5^ cells/well) were infected with various viruses at MOI of 0.01 for 8-16 h and analyzed by RT-qPCR for the expressions of downstream IL1β, IL8 and TNFα genes. ***p* < 0.01 vs NS controls. ^#^ *p* < 0.05, ^##^ *p* < 0.01 vs CRISPR controls. **(B)** The replications of GFP viruses including VSV, SeV and HSV1 were visualized under microscope.

Supplementary Figure 4 **to** Figures 7A–C No interactions between non-activated RLRs and between RLR and downstream MAVS. **(A)** pLGP2-FLAG and pRIG-I-HA, pLGP2-FLAG and pMDA5-HA, pRIG-I-HA and pMDA5-FLAG were co-transfected into 293T cells as in **Figures 7A–C** for 24 h. The cells lysates were immunoprecipitated with anti-HA mAb and subjected to Western-blotting using the indicated antibodies. **(B, C)** pLGP2-FLAG, pRIG-I-FLAG and pMDA5-FLAG (1.5 μg) were co-transfected into 293T cells with MAVS-GFP (1 μg) for 24 h, separately. Without **(B)** or with **(C)** EMCV (0.01 MOI) stimulation for 8h. The cells lysates were immunoprecipitated with anti-GFP mAb and subjected to Western-blotting using the indicated antibodies.

Supplementary Figure 5 **to** Figures 7D, E Co-localization of pLGP2 with pRIG-I and pMDA5 in activated 293T cells. **(A, B)** 293T cells grown on glass bottom cell culture dish (5×10^5^ cells) were co-transfected with pLGP2-HA (1 μg) and pRIG-I-FLAG (1.5 μg) or pLGP2-HA (1 μg) and pMDA5-FLAG (1.5 μg) for 24 h, then without **(A)** or with HMW poly I:C (1 μg/ml) transfection **(B)** for 8 h. The cells were examined for co-localization by con-focal fluorescence microscopy.
