# Supplementary figures and images for: Analysis of Porcine RIG-I Like Receptors Revealed the Positive Regulation of RIG-I and MDA5 by LGP2
Source: Front Immunol. 2021 May 18;12:609543. doi: 10.3389/fimmu.2021.609543 (PMC8169967; doi:10.3389/fimmu.2021.609543)

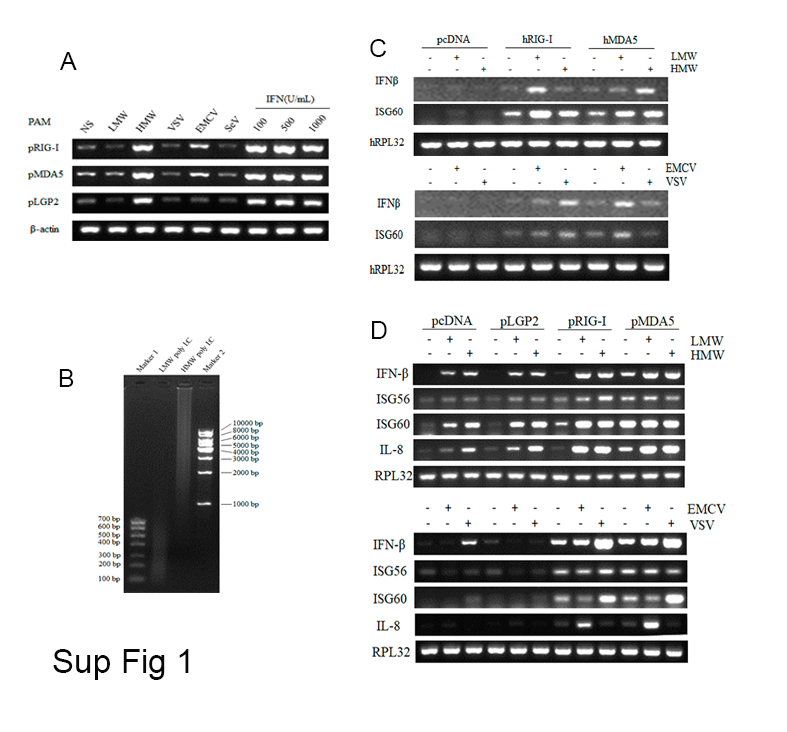

Supplement: Supplementary Figure 1 to Figure 2 — pRIG-I, pMDA5, and pLGP2 expressions can be induced by IFNβ and the activations of RIG-I and MDA5 by different agonists. (A) PAMs grown in 12-wells plates (5×10^5^ cells) were stimulated with various viruses (0.01 MOI each), LMW poly I:C transfection (1μg/ml), HMW poly I:C addition (10 μg/ml), or human IFNβ. The levels of porcine RLRs transcriptions were detected by RT-PCR. (B) The molecular weights of LMW and HMW poly I:C were detected by nucleic acid electrophoresis. (C) 293T cells grown in 24-well plates (3×10^5^ cells/well) were transfected with human RIG-I and MDA5 (0.5 μg each) using Lipofectamine 2000. Twenty-four hours post transfection, the cells were transfected with LMW poly I:C or HMW poly I:C (1 μg/ml) for 8 h (up), infected with 0.01 MOI EMCV or VSV for 8 h (down). The downstream genes were detected by RT-PCR as indicated. (D) 293T cells were transfected and stimulated as in Figure 2 , and the cells were analyzed by RT-PCR for the expressions of downstream genes. [file Image_1.tif]

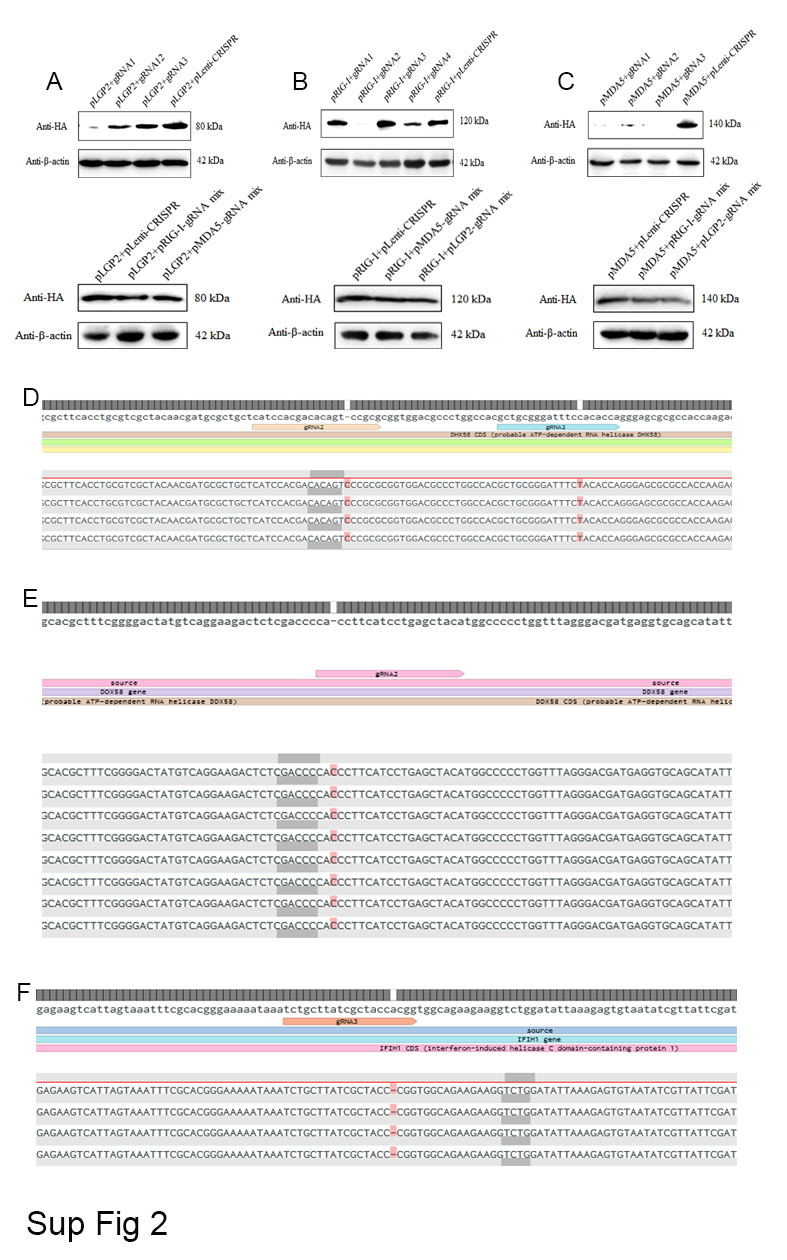

Supplement: Supplementary Figure 2 to Figures 4, 5 — The efficacy and specificity of CRISPR gRNAs and the verification of homozygous KO PAM cell clones. (A) The pLGP2-HA were co-transfected into 293T cells with gRNAs designed for pLGP2, pRIG-I and pMDA5. (B) The pRIG-I-HA were co-transfected into 293T cells with gRNAs designed for RIG-I, pMDA5 and pLGP2. (C) The pMDA5-HA were co-transfected into 293T cells with gRNAs designed for pMDA5, pRIG-I and pLGP2. The HA tagged pLGP2, pRIG-I and pMDA5 were analyzed by Western-blotting. (D–F) The genome DNA regions covering the gRNAs were amplified by PCR using the primers as shown in Supplementary Table 2 and the PCR products of individual PAM clones of pLGP2 (D), pRIG-I (E) and pMDA5 (F) were cloned into T vectors and sequenced for base insertion/deletion mutations. [file Image_2.tif]

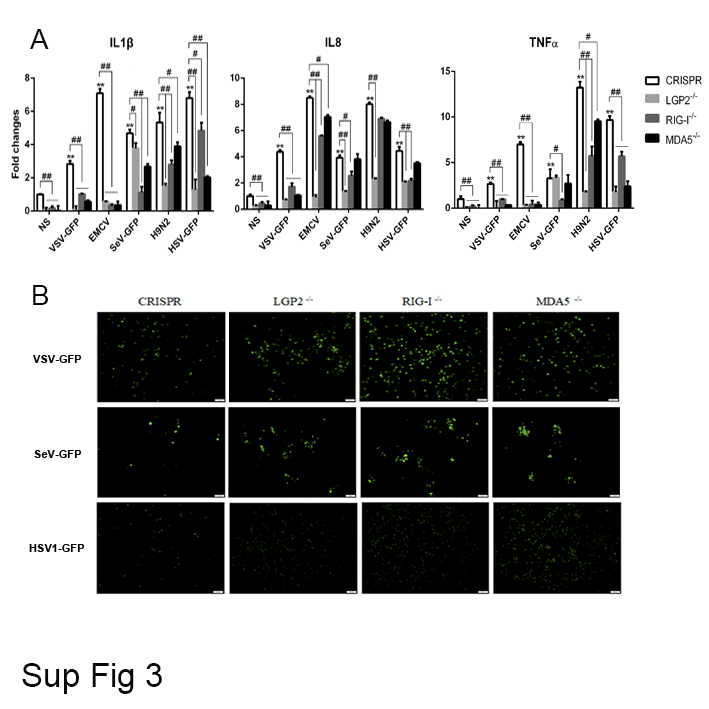

Supplement: Supplementary Figure 3 to Figures 5C, D — (A) Expression of downstream genes in LGP2-/-, RIG-/- and MDA5-/- PAMs infected with various viruses. The LGP2-/-, RIG-I-/- and MDA5-/- PAMs plus CRISPR control PAMs in 12-well plates (5×10^5^ cells/well) were infected with various viruses at MOI of 0.01 for 8-16 h and analyzed by RT-qPCR for the expressions of downstream IL1β, IL8 and TNFα genes. **p < 0.01 vs NS controls. # p < 0.05, ## p < 0.01 vs CRISPR controls. (B) The replications of GFP viruses including VSV, SeV and HSV1 were visualized under microscope. [file Image_3.tif]

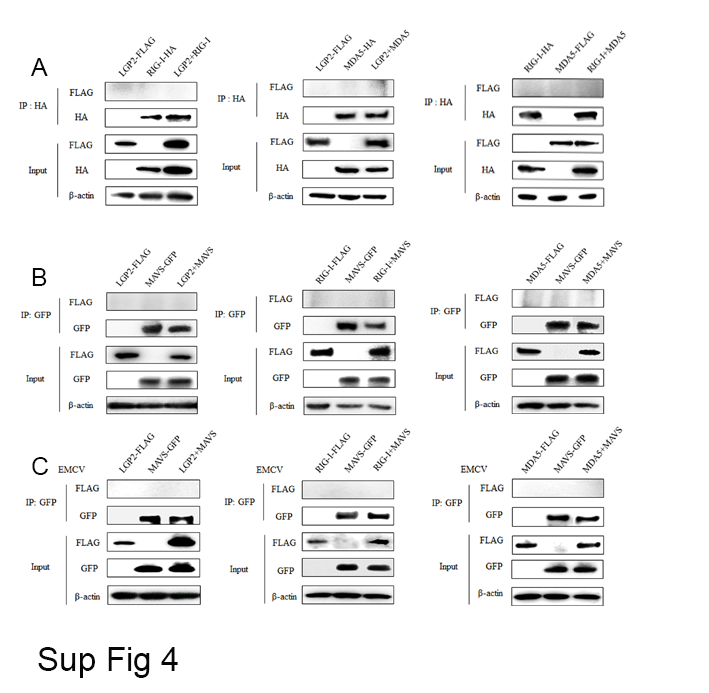

Supplement: Supplementary Figure 4 to Figures 7A–C — No interactions between non-activated RLRs and between RLR and downstream MAVS. (A) pLGP2-FLAG and pRIG-I-HA, pLGP2-FLAG and pMDA5-HA, pRIG-I-HA and pMDA5-FLAG were co-transfected into 293T cells as in Figures 7A–C for 24 h. The cells lysates were immunoprecipitated with anti-HA mAb and subjected to Western-blotting using the indicated antibodies. (B, C) pLGP2-FLAG, pRIG-I-FLAG and pMDA5-FLAG (1.5 μg) were co-transfected into 293T cells with MAVS-GFP (1 μg) for 24 h, separately. Without (B) or with (C) EMCV (0.01 MOI) stimulation for 8h. The cells lysates were immunoprecipitated with anti-GFP mAb and subjected to Western-blotting using the indicated antibodies. [file Image_4.tif]

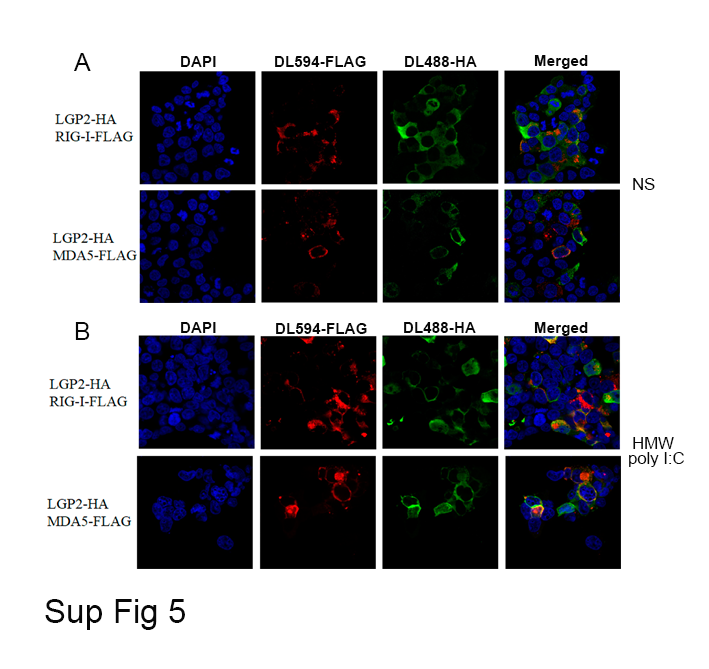

Supplement: Supplementary Figure 5 to Figures 7D, E — Co-localization of pLGP2 with pRIG-I and pMDA5 in activated 293T cells. (A, B) 293T cells grown on glass bottom cell culture dish (5×10^5^ cells) were co-transfected with pLGP2-HA (1 μg) and pRIG-I-FLAG (1.5 μg) or pLGP2-HA (1 μg) and pMDA5-FLAG (1.5 μg) for 24 h, then without (A) or with HMW poly I:C (1 μg/ml) transfection (B) for 8 h. The cells were examined for co-localization by con-focal fluorescence microscopy. [file Image_5.tif]
