# Supplementary material for: Analysis of Porcine RIG-I Like Receptors Revealed the Positive Regulation of RIG-I and MDA5 by LGP2
Source: Front Immunol. 2021 May 18;12:609543. doi: 10.3389/fimmu.2021.609543 (PMC8169967; doi:10.3389/fimmu.2021.609543)
Supplement: Supplementary file 6 [file Table_1.docx]

Supplementary Table 1: The PCR primers used for gene cloning and mutations

| **Primer names** | **Primer sequences** |
| --- | --- |
| pLGP2 | F: ttcgcGTCGACatggagctgcgaccctaccagtg |
|  | R: ttcgcGATATCgtccagggagaggtcggtcagg |
| pRIG-I | F: ttcgcGTCGACatgacagcagagcagcggcggaatc |
|  | R:ttcgcCCCGGGctcaaggttgcccattccctgaagacccag |
| pMDA5 | F: ttcgcGTCGACatgtcgtcggatgggtattccgcg |
|  | R: ttcgcCCCGGGgtcctcatcactagacaaacaatattctgaatagttaagatcggg |
| L1 | F: cccgcGCTAGC atggagctgcgaccctac |
|  | R: cccgcGATATC gggtgacatgatgcgcc |
| L∆1 | F (L2 F): cccgcGCTAGCcgcctggcagctgatg |
|  | R (L3 R): ctagctggtGATATCgtccagg |
| L2 | F: cccgcGCTAGCcgcctggcagctgatg |
|  | R: cccgcGATATCccgctgcagctcctgg |
| L∆2 | F (L1 F): cccgcGCTAGC atggagctgcgaccctac |
|  | R (L1 R): cccgcGATATC gggtgacatgatgcgcc |
|  | F (L3 F): cccgcGCTAGCgcagccttggttaagcgg |
|  | R (L3 R): ctagctggtGATATCgtccagg |
|  | Fusion primer: gcgcatcatgtcacccgcagccttggttaagcggg |
| L3 | F: cccgcGCTAGCgcagccttggttaagcgg |
|  | R: ctagctggtGATATCgtccagg |
| L∆3 | F (L1 F): cccgcGCTAGC atggagctgcgaccctac |
|  | R (L2 R): cccgcGATATCccgctgcagctcctgg |
| LGP2 K30A | F: gccgccctggtcgcgccagcgcccgt |
|  | R: acgggcgctggcgcgaccagggcggc |
| LGP2 K138E / Y142F | F: gctcaggatgatgttgaagacggtgtcctcgtgtgtgtggtgacac |
|  | R: gtgtcaccacacacacgaggacaccgtcttcaacatcatcctgagc |
| LGP2 T167A / S169A | F: gtgcctggggcggctgcgagcccgagc |
|  | R: gctcgggctcgcagccgccccaggcac |
| LGP2 K654E R | F: gggcacgcaggaccactccttgacctggatccg |
|  | R: cggatccaggtcaaggagtggtcctgcgtgccc |

Supplementary Table 2: The CRISPR gRNA encoding sequences and PCR primers for porcine LGP2, RIG-I, MDA5 genes

| **gRNA names** | **gRNA encoding DNA sequences** |
| --- | --- |
| pLGP2 gRNA1 | F: CACCgcgatgacccagatgacccag |
|  | R: AAACctgggtcatctgggtcatcgc |
| pLGP2 gRNA2 | F: CACCgcatccacgacacagtccgcg |
|  | R: AAACcgcggactgtgtcgtggatgc |
| pLGP2 gRNA3 | F: CACCgctgcgggatttccacacca |
|  | R: AAACtggtgtggaaatcccgcagc |
| pRIG-I gRNA1 | F: CACCgcgacgagttgggaaatggg |
|  | R: AAACcccatttcccaactcgtcgc |
| pRIG-I gRNA2 | F: CACCgtgtagctcaggatgaaggtg |
|  | R: AAACcaccttcatcctgagctacac |
| pRIG-I gRNA3 | F: CACCgagaagaaatgaaaccgcgct |
|  | R: AAACagcgcggtttcatttcttctc |
| pRIG-I gRNA4 | F: CACCgcaggctgagaaaaacaacaa |
|  | R: AAACttgttgtttttctcagcctgc |
| pMDA5 gRNA1 | F: CACCggaacgatgatggtgcacaa |
|  | R: AAACttgtgcaccatcatcgttcc |
| pMDA5 gRNA2 | F: CACCgaacatcctagtccagccggg |
|  | R: AAACcccggctggactaggatgttc |
| pMDA5 gRNA3 | F: CACCgtctgcttatcgctaccacgg |
|  | R: AAACccgtggtagcgataagcagac |
|  | **PCR primer sequences** |
| LGP2 gRNA 1 PCR | F: gactgtggacatcagggcc |
|  | R: ttgcactgggggatgtcca |
| LGP2 gRNA 2-3 PCR | F: agcagcaagtggtggagct |
|  | R: gccagctcattcttgtgatcatca |
| RIG-I gRNA 2, 4 PCR | F: atgacagcagagcagcgg |
|  | R: atccaaaaagcctcggaacca |
| MDA5 gRNA 1 PCR | F: ctgactatcaaacaaatggcgaga |
|  | R: tagcgggccgctaaggagt |
| MDA5 gRNA 2 PCR | F: tgattggagctggacacagc |
|  | R: ctccagagccactttgggcaa |
| MDA5-gRNA3 PCR | F: agattcagagggcggtcgc |
|  | R: ggaaatgtgatgggtaattctaccc |
| T vector universal primer | M13 F: tgtaaaacgacggccagt  M13 R: caggaaacagctatgacc |

Supplementary Table 3: Primers for RT-PCR and RT-qPCR in this study

| **Primer names** | **Primer sequences** |
| --- | --- |
| hIFN-β | F: tgggaggattctgcattacc |
|  | R: cagcatctgctggttgaaga |
| hISG56 | F: cgctatagaatggagtgtcca |
|  | R: tttcctccacacttcagca |
| hISG60 | F: agtctagtcacttggggaaac |
|  | R: ataaatctgagcatctgagagtc |
| hIL-8 | F: gtttttgaagagggctgagaattc |
|  | R: catgaagtgttgaagtagatttgcttg |
| hRPL32* | F: caacattggttatggaagcaaca |
|  | R: tgacgttgtggaccaggaact |
| pIFN-β | F: tgagcattctgcagtacctga |
|  | R: ccggaggtaatctgtaagtctgt |
| pISG56 | F: atgggagttggtcattcaaga |
|  | R: caggtgtttcacataggcca |
| pISG60 | F: cccgacaacccagaattctcct |
|  | R: agagcgctgatgaagttgttgc |
| pIL-1β | F: agggacatggagaagcgat |
|  | R: ttctgcttgagaggtgctga |
| pIL-8 | F: ctgcagttctggcaagagtaagt |
|  | R: cactctcaatcactctcagttcct |
| pTNF-α | F: atcgccgtctcctaccaga |
|  | R: tcgatcatccttctccagct |
| pLGP2 | F: ccaccaagacccagatccta |
|  | R: gacccttgaactgcttctgc |
| pRIG-I | F: cccagtgtatgagcagcaga |
|  | R: ctggtgttgtggcattcatc |
| pMDA5 | F: ctcaaagagcatcccctgag  R: gttcgaactctttgcggaag |
| pTLR3 | F: ggtactgttgcccttttgga |
|  | R: aattctggctccagctttga |
| pβ-actin* | F: atgaagatcaagatcatcgcg |
|  | R: tcgtactcctgcttgctgatc |
| MIFN-β | F: aaattgctctcctgttgtgcttct |
|  | R: aagccttccattcaattgcca |
| MIL-1β | F: ccagtgaaatgatggcttac |
|  | R: gctacaacaaccgacacg |
| MIL-8 | F: ttcctgctttctgcagctctgt |
|  | R: tggtccactctcaatcactctcagt |
| MTNF-α | F: catgttgtagcaaaccctcaagc |
|  | R: atggcaccaccagctggttat |
| Mβ-actin* | F: agaagatgacccagatcatgtttga |
|  | R: tccatcacgatgccagtggta |
| SIV H9N2 HA | F: aaaccaatgatagggccaa |
|  | R: ttgcactacacagttaccac |
| SIV H9N2 M | F: gaccratcctgtcacctctgac |
|  | R: agggcattytggacaaakcgtcta |
| HSV1 gB | F: ttctgcagctcgcaccac |
|  | R: ggagcgcatcaagaccacc |
| VSV Glycoprotein | F: gaggagtcacctggacaatcact |
|  | R: tgcaaggaaagcattgaacaa |
| EMCV Polyprotein | F: tcaccgtgaagtccggcagt |
|  | R: tgtcagacgctgtggcctga |
| PEDV M | F: ggacacattcttggtggtct |
|  | R: cgccagtagcaaccttatagccctcta |
| PRRSV N | F: ataacaacggcaagcagcag |
|  | R: ctctggactggttttgttg |
| SeV GFP | F: gcaacatcctggggcacaagct |
|  | R: cgcgcttctcgttggggtcttt |

Note: h denotes human; p denotes porcine; M denotes monkey. * refers to the housekeeping genes.
